# Novel amide-based macrocyclic Co(ii) complexes: correlating structural, computational, and biological properties through DFT and docking

**DOI:** 10.1039/d5ra08873a

**Published:** 2026-01-14

**Authors:** Subhash, Manish Kumar, Vandana, Annu Devi, Ashu Chaudhary

**Affiliations:** a Department of Chemistry, Smt. Devkiba Mohansinhji Chauhan College of Commerce and Science, UT of DNH & DD Silvassa 396230 India; b Department of Chemistry, Maharshi Dayanand University Rohtak 124001 Haryana India; c Department of Chemistry, Kurukshetra University Kurukshetra 136119 Haryana India ashuchaudhary21@gmail.com ashuchaudhary@kuk.ac.in +91-9729864551

## Abstract

A series of Co(ii) complexes incorporating functionalized macrocyclic scaffolds, designated as [Co(N_4_O_4_MacL_1_)Cl_2_]–[Co(N_4_O_4_MacL_3_)Cl_2_], were synthesized using three structurally related macrocyclic ligands (N_4_O_4_MacL_1_–N_4_O_4_MacL_3_), each featuring an amide-based tetradentate donor framework. The complexes were comprehensively characterized by elemental analysis, FTIR spectroscopy, magnetic susceptibility measurements, UV-visible spectroscopy, ^1^H and ^13^C NMR spectroscopy, and powder X-ray diffraction. Spectroscopic and analytical findings confirm that the ligands act as monoanionic tetradentate donors, coordinating through nitrogen and oxygen donor sites. The magnetic and electronic spectral data support an octahedral coordination geometry around the Co(ii) center in all complexes. Thermal stability and kinetic parameters were assessed through thermogravimetric analysis, employing the Coats-Redfern and Flynn-Wall-Ozawa (FWO) models to determine activation energies and thermodynamic parameters. Moreover, density functional theory (DFT) calculations provided insights into optimized geometries, bond parameters, and frontier molecular orbital characteristics, which corroborate the experimental observations. The biological activities of the ligands and their Co(ii) complexes were evaluated through *in vitro* antimicrobial assays against fungal strains *A. niger* and *C. albicans*, and bacterial strains *B. subtilis*, *S. aureus*, *E. coli*, and *S. typhi*. Additionally, antioxidant activity assessed by the DPPH free-radical scavenging assay revealed that [Co(N_4_O_4_MacL_3_)Cl_2_] demonstrated the highest radical-quenching efficiency. Among the complexes studied, [Co(N_4_O_4_MacL_2_)Cl_2_] displayed the most promising antimicrobial activity with minimal cytotoxic effects. Molecular docking studies further validated the biological results, indicating favourable interactions of the complexes with relevant biomolecular targets. Overall, these findings highlight the therapeutic potential of amide-containing macrocyclic ligands and their cobalt(ii) complexes as promising candidates for antimicrobial applications, selective cytotoxic behaviour, and effective antioxidant activity.

## Abstract 2

Amide-based macrocyclic Co(ii) complexes with octahedral geometry were synthesized and characterized by spectral, thermal and DFT studies. The complexes showed enhanced antimicrobial and antioxidant activity, supported by docking results.

## Graphical abstract

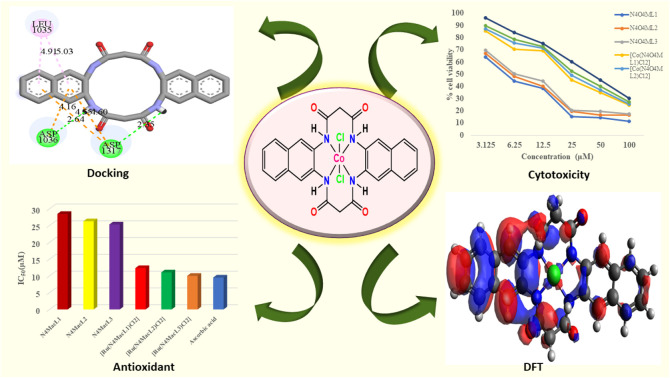

## 1 Introduction

Bioinorganic chemistry has emerged as an important and expanding branch of inorganic chemistry, tracing its origins to the development of cisplatin one of the first metal-based anticancer drugs. Cancer, characterized by the uncontrolled proliferation of abnormal cells, leads to life-threatening diseases. As reported by the World Health Organization, it stands as the second most common cause of illness and death worldwide, following cardiovascular diseases.^1^ Although significant advancements have been made in cancer detection, treatment, and prevention, it continues to be a leading cause of mortality across the globe. Most cancer drugs currently in use are not specifically targeted toward tumour cells; instead, they exhibit general cytotoxic effects that also harm healthy cells. Consequently, there is an increasing need to design new and more selective therapeutic agents for cancer treatment.^2–4^ The search for novel compounds with antitumor potential remains a crucial focus of pharmaceutical research. Metal complexes containing oxygen- and nitrogen-donor ligands have shown promising biological activities, including antibacterial, antifungal, and antitumor effects, along with various biochemical, pharmacological, and clinical properties.^5–10^ These biologically active substances include metal ions, coordination compounds, complex ions, and other inorganic molecules.^11,12^ The strong chelating ability of macrocyclic ligands plays an important role in their antioxidant behaviour, which can contribute to the development of transition-metal-based anticancer agents.^13–16^ Moreover, density functional theory (DFT) has become an increasingly valuable tool in theoretical studies of metal complexes, providing insights into their stereochemical structures and electronic properties.^17–20^ The remarkable biological responsiveness of macrocyclic compounds and their ability to chelate transition metal ions present in biological systems have attracted significant scientific attention. These compounds exhibit strong chelating tendencies, allowing them to form a wide range of coordination geometries from bidentate to multidentate through donor systems such as ONO, and NO. Metal complexes of transition metals including Mn(ii), Cu(ii), Cr(iii), and Fe(iii) have demonstrated notable roles in biological processes, particularly exhibiting antioxidant, antimicrobial, DNA-binding, and cytotoxic properties.^21–24^ Consequently, macrocyclic ligands containing the donor framework (N_4_O_4_MacL_1_–N_4_O_4_MacL_2_) and their corresponding metal complexes are anticipated to possess significant and diverse biological activities.

## 2 Experimental section

### 2.1 Material and methods

All culture media utilized in this study were procured from HiMedia (India) and used as supplied, without additional processing. High-purity analytical-grade solvents were employed directly, with no further purification steps. Elemental composition of the synthesized compounds was determined using an Elementar Vario EL III CHN analyzer at the Sophisticated Analytical Instrumentation Facility (SAIF). Fourier-transform infrared (FT-IR) spectra were recorded on a PerkinElmer Spectrum IR (Version 10.6.2) spectrophotometer using KBr pellets prepared in-house. Electron paramagnetic resonance (EPR) analyses were performed in both solid state and DMF medium at liquid nitrogen temperature on a Varian F-113 X-band spectrometer, employing TCE (*g* = 2.00277) as the ordinary SAIF, at, IIT Bombay, India. The cancer cell line employed in this investigation was kindly provided by a laboratory at the Indian Institute of Science (IISc), Bengaluru, India, and was maintained under standard culture conditions. Proton and carbon NMR spectra were obtained in DMSO-*d*_6_ using a JEOL 400 ECZS spectrometer. Electron-ionization mass spectra (EI-MS) for the synthesized ligands and cobalt(ii) complexes were acquired on a XEVO G2-XS QTof high-resolution mass spectrometer to validate molecular weights and assess characteristic fragmentation behavior. UV-visible spectral measurements were carried out in DMSO using a PerkinElmer Lambda series UV-vis spectrophotometer. Powder X-ray diffraction (PXRD) data were collected on a PANalytical X'Pert Pro diffractometer. Thermal behavior and decomposition characteristics were evaluated *via* thermogravimetric analysis (TGA) using a Shimadzu Model 50 instrument.

### 2.2 Design and execution of synthetic pathways

#### 2.2.1 Preparation of N_4_O_4_-based macrocyclic ligands (N_4_O_4_MacL_1_–N_4_O_4_MacL_3_)

The Macrocyclic ligand N_4_O_4_MacL_1_ was prepared *via* a template-free condensation reaction. A stoichiometric mixture of dicarboxylic acid (2.082 g, 0.02 mol) and 1,8-diaminonaphthalene (3.162 g, 0.02 mol) dissolved in ethanol (20 mL each) was heated to reflux with continuous stirring for ∼8 hours, facilitating macrocyclic ring closure *via* amide bond formation. A few drops of concentrated hydrochloric acid were introduced to facilitate protonation and promote cyclization through amide bond formation. During reflux, gradual color deepening and increased viscosity were observed, indicating the progression of cyclocondensation. Upon completion, the reaction mixture was allowed to cool to room temperature, yielding a white precipitate. The solid product was filtered, repeatedly washed with chilled ethanol to remove unreacted precursors and side-products, and subsequently dried under vacuum over phosphorus pentoxide (P_4_O_10_) to afford the ligand in pure form. Following this optimized protocol, ligands N_4_O_4_MacL_2_ and N_4_O_4_MacL_3_ were synthesized by replacing malonic acid with butane-1,4-dioic acid (2.318 g, 0.02 mol) and pentane-1,5-dioic acid (2.629 g, 0.02 mol), respectively. Increasing the methylene linkers in the dicarboxylic acid precursors allowed systematic control over ring size and ligand flexibility, enabling a series of structurally related macrocycles.^25^

#### 2.2.2 Metallation of macrocyclic ligands: cobalt(ii) complexes [Co(N_4_O_4_MacL_1_)Cl_2_]–[Co(N_4_O_4_MacL_3_)Cl_2_]

The cobalt(ii) macrocyclic complex [Co(N_4_O_4_MacL_1_)Cl_2_] was obtained through a direct metal–ligand coordination reaction.^26^ A hot ethanolic solution of cobalt(ii) chloride hexahydrate (CoCl_2_·6H_2_O) (0.4760 g, 0.002 mol) was added gradually to an equimolar ethanolic solution (20 mL) of the ligand N_4_O_4_MacL_1_ (0.9047 g, 0.002 mol) under vigorous stirring. The mixture was heated under reflux at 54–66 °C for approximately 7–11 h to ensure complete complexation. As the reaction progressed, a noticeable color change and enhanced turbidity indicated the formation of the cobalt complex. After cooling to room temperature, the resulting solid precipitate was isolated by filtration, thoroughly washed with chilled ethanol to remove unbound ligand or metal salt residues, and dried under vacuum over phosphorus pentoxide (P_4_O_10_) to obtain the purified product.

**Scheme 1:**
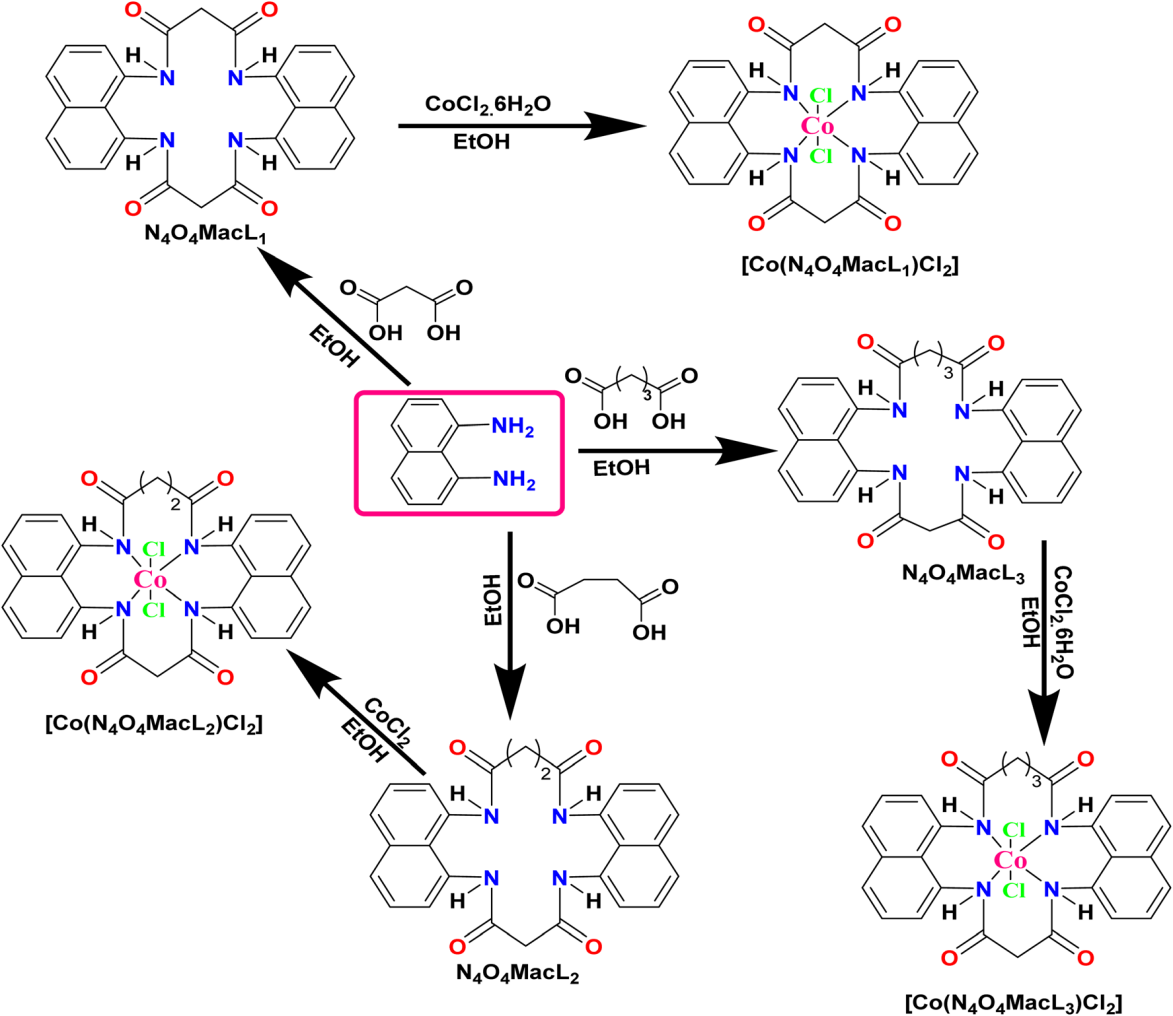
Schematic representation of the synthesis of macrocyclic ligands (N_4_O_4_MacL_1_–N_4_O_4_MacL_3_) and their corresponding Co(ii) complexes.

Following the same optimized protocol, cobalt complexes [Co(N_4_O_4_MacL_2_)Cl_2_] and [Co(N_4_O_4_MacL_3_)Cl_2_] were synthesized by substituting N_4_O_4_MacL_1_ with N_4_O_4_MacL_2_ (0.9615 g, 2 mmol) and N_4_O_4_MacL_3_ (1.0159 g, 2 mmol), respectively. Variation in ligand chain length facilitated controlled modulation of the macrocyclic cavity and metal coordination environment. The successful formation of the complexes was confirmed by characteristic spectroscopic features, including ligand-to-metal charge transfer bands and disappearance of free ligand functional group signals. To ensure high purity of the synthesized compounds, we adopted a combination of careful synthetic control and multiple purification steps. High purity levels were primarily achieved by optimizing reaction conditions, including reagent molar ratios, solvent selection, reaction time, and temperature, which minimized the formation of side products. After completion of the reaction, the crude product was subjected to appropriate purification techniques such as recrystallization, solvent–solvent extraction, and column chromatography depending on the compound's physicochemical properties. These steps ensured selective isolation of the target compound (Scheme 1).

### 2.3 DFT calculations

Quantum-chemical investigations of the tetra amide based macrocyclic ligands (N_4_O_4_MacL_1_–N_4_O_4_MacL_3_) and their cobalt(ii) complexes were performed using the ORCA computational suite^27^ employing the density functional theory (DFT) framework. Geometry optimizations were conducted without imposing symmetry constraints.^28^ The hybrid B3LYP functional was used in combination with the def2-SVP basis set for ligand atoms, while the def2-TZVP basis set was applied to cobalt centers to ensure reliable treatment of transition-metal electronic environments. The B3LYP functional was selected because it is one of the most extensively benchmarked hybrid functionals for geometry optimization, electronic structure calculations, and prediction of molecular properties of coordination and organic compounds. Numerous studies demonstrate that B3LYP provides a reliable balance between computational cost and accuracy for systems comparable to those investigated in this work. The def2-SVP basis set was employed as it offers a well-validated split-valence description with polarization functions, enabling accurate geometry optimization while maintaining reasonable computational efficiency for molecules of moderate size. Test calculations using a larger def2-TZVP basis set on representative structures showed only marginal differences in optimized geometries and electronic energies, supporting the suitability of def2-SVP for full-scale calculations. Regarding dispersion and solvent corrections, the primary focus of our study was on gas-phase intrinsic electronic properties, for which B3LYP/def2-SVP provides dependable results. Nonetheless, dispersion-corrected (D3) and implicit solvent (CPCM) single-point energy calculations were additionally performed on the optimized geometries to verify the robustness of the computed parameters.

After successful optimization, no imaginary frequencies were observed, confirming that the structures correspond to true energy minima. Frontier molecular orbital energies, including the highest occupied molecular orbital (HOMO) and lowest unoccupied molecular orbital (LUMO), total electronic energies, and dipole moments were calculated to assess electronic characteristics and reactivity profiles. In addition, optimized geometrical parameters such as selected bond lengths, bond angles, and coordination metrics around the metal ion were examined to elucidate structural features and intra-ligand interactions. These theoretical insights complement the experimental findings, providing a deeper understanding of electronic distribution, stability, and molecular geometry in both the free ligands and their cobalt(ii) complexes.

### 2.4 Biology

#### 2.4.1 Antimicrobial action

The antibacterial properties of the synthesized ligands and their cobalt(ii) complexes were assessed by the agar disc diffusion method^29^ against selected Gram-positive (*Bacillus subtilis* and *Staphylococcus aureus*) and Gram-negative (*Escherichia coli* and *Salmonella typhi*) bacterial strains.

Nutrient agar medium was prepared using peptone (5 g), beef extract (5 g), sodium chloride (5 g), and agar–agar (20 g) in 1000 mL of distilled water. The sterilized medium was poured into Petri plates and allowed to solidify. Subsequently, ∼5.4 mL of sterile molten nutrient agar inoculated with the test culture (approximately 41 °C) was layered over the base medium to obtain a uniform lawn of microbial growth. Test samples were dissolved in petroleum ether to obtain concentrations of 30 and 50 ppm. Sterile Whatman no. 1 filter paper discs (5 mm diameter) were impregnated with the sample solutions, air-dried under aseptic conditions, and carefully placed on the inoculated agar plates. The plates were incubated at 29.4 °C for 22 h, after which the diameters of inhibition zones (in mm) were recorded to evaluate antibacterial activity. Ampicillin served as the positive control and was tested under identical conditions using the same solvent system. The antifungal efficacy of ligands (N_4_O_4_MacL_1_–N_4_O_4_MacL_3_) and their corresponding cobalt(ii) complexes ([Co(N_4_O_4_MacL_1_)Cl_2_]–[Co(N_4_O_4_MacL_3_)Cl_2_]) was examined using the agar well diffusion method^29^ against pathogenic fungi *Aspergillus niger* and *Candida albicans*. Experimental conditions including medium composition, inoculum preparation, sample concentrations, and incubation parameters were maintained similar to the antibacterial protocol to ensure consistency. Zones of fungal growth inhibition were measured to determine antifungal potential.

### 2.5 Antioxidant activity

The antioxidant efficiency of the synthesized ligands and their cobalt(ii) complexes was assessed using the DPPH (2,2-diphenyl-1-picrylhydrazyl) free-radical scavenging method. The assay was conducted spectrophotometrically using a UV-visible spectrophotometer. A 1 mM DPPH stock solution was freshly prepared in methanol,^30^ and serial dilutions of the test samples (0–60 mg mL^−1^) were prepared in DMSO. Ascorbic acid served as the reference antioxidant under identical experimental conditions.

For each experiment, 1.5 mL of the test solution was combined with 3.5 mL of 0.1 mM DPPH solution in methanol. The reaction mixtures were vortexed briefly to ensure homogenization and incubated at room temperature in the dark for 30 minutes to allow complete interaction between the radical species and the test compounds. A control was prepared using DPPH solution without the sample. Following incubation, absorbance values were measured at 517 nm, corresponding to the characteristic absorbance maximum of DPPH. The decrease in absorbance relative to the control indicated free-radical scavenging capacity.^31^

The experimental protocol enabled quantitative determination and comparison of radical-scavenging performance among the synthesized ligands and their cobalt(ii) derivatives.

### 2.6 Cytotoxicity

The *in vitro* cytotoxic potential of the synthesized Macrocyclic ligands (N_4_O_4_MacL_1_–N_4_O_4_MacL_3_) and their corresponding cobalt(ii) complexes was evaluated against human breast carcinoma (MCF-7) and hepatocellular carcinoma (HepG-2) cell lines using the standard MTT colorimetric assay.^32^ MCF-7 and HepG-2 cells were routinely maintained in Dulbecco's Modified Eagle Medium (DMEM) supplemented with 10% fetal bovine serum (FBS), 1% penicillin-streptomycin (P/S), and incubated at 37 °C in a humidified atmosphere containing 5% CO_2_. Cells were seeded into sterile 96-well plates at a density of approximately 1 × 10^4^ cells per well and allowed to adhere overnight. Upon reaching ∼92–94% confluence, cells were treated with graded concentrations of the synthesized compounds (3.125–100.0 µM) along with colchicine, which served as a positive control. Untreated cells incubated under identical conditions were used as a negative control. Plates were then incubated for 46 h at 38 °C to allow compound cell interaction. All experiments were conducted in triplicate and results were expressed as mean ± standard error (SE). The cytotoxicity dose–response curves were generated using a four-parameter logistic (4 PL) non-linear regression model,^33^ which is widely accepted for sigmoidal dose–response analyses. The model is defined as:*Y* = Bottom + (Top − Bottom)/[1 + (*X*/IC_50_)^*n*^]where *Y* represents the percentage cell viability, *X* is the tested concentration, Top and Bottom are the upper and lower asymptotes of the curve, IC_50_ is the concentration that reduces viability by 50%, and *n* denotes the Hill slope of the curve.

### 2.7 Molecular docking investigations

The Molecular docking has recently become a vital tool in the design and development of new drug candidates.^34^ Its primary purpose is to elucidate drug–nucleic acid or drug–protein interactions and to predict the preferred binding orientation of a ligand within a target biomolecule. In this study, AutoDock Vina,^35^ a rigid docking software, was employed to investigate the binding interactions of three Macrocyclic ligands (N_4_O_4_MacL_1_–N_4_O_4_MacL_3_). The crystal structures of all target receptors were obtained from the Protein Data Bank.

The study aimed to predict the most favorable binding modes and the corresponding lowest binding energies of the ligands. Experimental results indicated that N_4_O_4_MacL_2_ exhibited notable *in vitro* antibacterial and antifungal activity, effectively inhibiting the growth of bacteria and fungi. Docking analyses revealed that all three ligands (N_4_O_4_MacL_1_–N_4_O_4_MacL_3_) showed significant interactions with proteins from *E. coli*, *S. aureus*, *C. albicans*, and *B. subtilis*. The binding energies (kcal mol^−1^) calculated at the active sites of these organisms provided a qualitative assessment of inhibitory potentimagal.

Notably, N_4_O_4_MacL_2_, which contains a para-substituted halogen on the benzene ring, exhibited stronger binding to *E. coli* proteins compared to streptomycin, suggesting that this substitution enhances the ligand's fit within the active site and contributes to its biological activity.^36^

## 3 Results and discussion

### 3.1 Common features

The physical, analytical, and spectroscopic characteristics of the ligands (N_4_O_4_MacL_1_–N_4_O_4_MacL_3_) and their divalent cobalt complexes are summarized in the experimental section (Table S1). The complexes are soluble in polar aprotic solvents such as DMF and DMSO and exhibit stability under ambient conditions. The experimentally determined elemental composition closely matches the calculated values, confirming the formation of the complexes in a 1 : 1 metal-to-ligand stoichiometry.^37^

### 3.2 FT-IR characterization and metal–ligand binding features

**Fig. 1:**
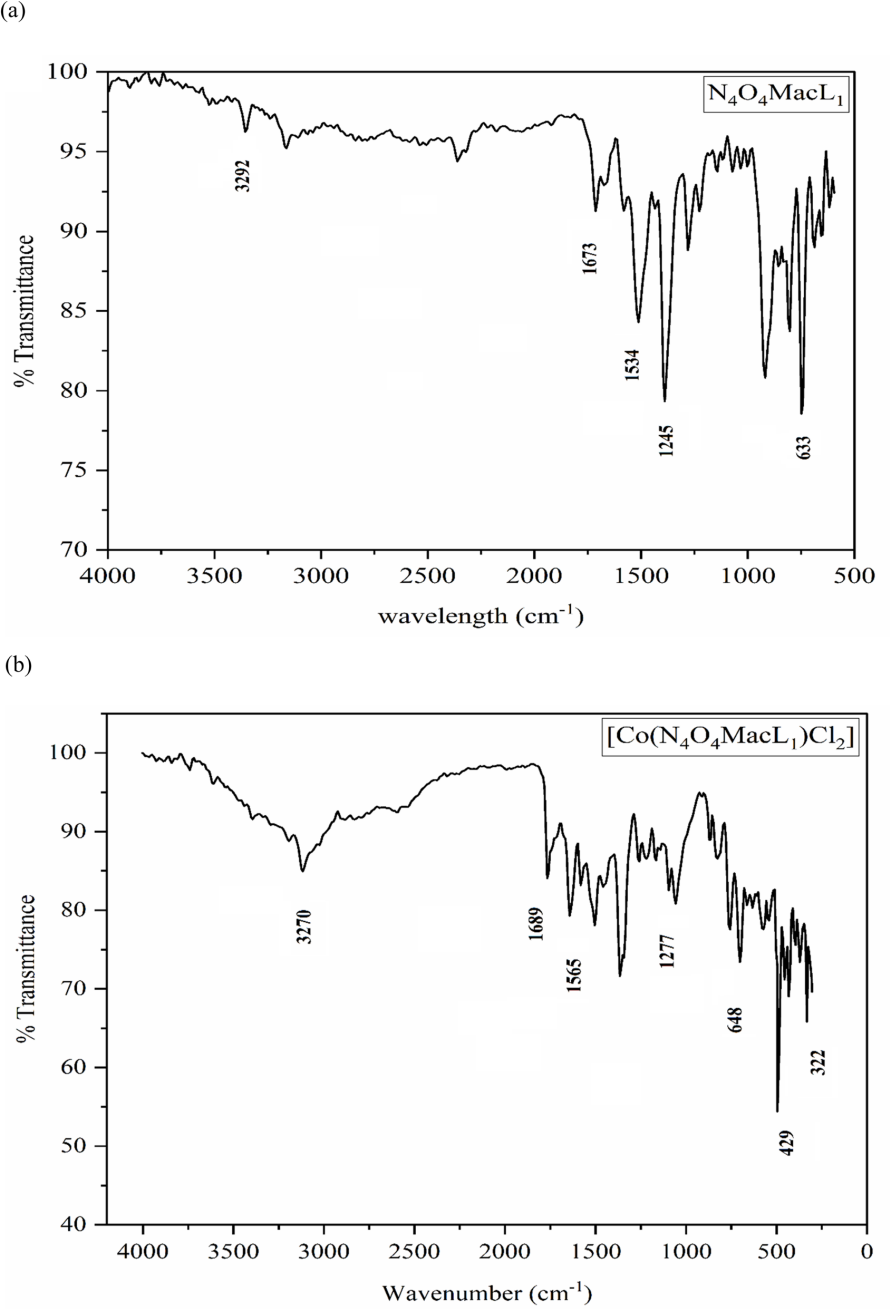
Infra-red spectra (a) N_4_O_4_MacL_1_ (b) [Co(N_4_O_4_MacL_1_)Cl_2_].

The FT-IR spectra of the complexes provided the first indication of successful macrocyclic complex formation, as several characteristic peaks present in the spectra of the free ligands were absent in the complexes. The sharp band observed in the 3289–3302 cm^−1^ range in the ligand spectra was assigned to the cyclized, non-coordinated amide N–H stretch.^38^ Strong absorption bands between 1536–1582 cm^−1^, formed through an intramolecular condensation reaction between the –OH groups of the dicarboxylic acids and the –NH_2_ moieties of 1,8-diaminonaphthalene, leading to macrocyclic ring closure, further supported the formation of macrocyclic ligands. Additionally, four amide-associated bands appeared at 1663–1673, 1534–1552, 1245–1279, and 633–649 cm^−1^, consistent with those reported for previously characterized macrocyclic compounds (Fig. 1).^39^

In the cobalt complexes, the amide band shifted slightly to 1679–1689 cm^−1^ compared to 1663–1673 cm^−1^ in the free ligands, indicating coordination *via* the nitrogen atoms of the macrocyclic moiety (Fig. S1–S3).^41^ A new band observed in the 429–455 cm^−1^ region was assigned to the *ν*(M–N) vibration, confirming metal–nitrogen coordination.^40^ Furthermore, bands appearing at 322–343 cm^−1^ were attributed to *ν*(M–Cl) vibrations (Table 1). The combined spectral signatures demonstrate that cobalt(ii) is hexacoordinated through four amide nitrogen donors and two coordinating chloride anions.

**Table 1:** FT-IR bands for ligands and Co(ii) complexes (cm^−1^)

Complexes	*ν*(–NH)	Amide				*ν*(Co–N)	*ν*(Co–Cl)
		I	II	III	IV		
[N_4_O_4_MacL_1_]	3292	1673	1534	1245	633	—	—
[N_4_O_4_MacL_2_]	3284	1668	1546	1269	638	—	—
[N_4_O_4_MacL_3_]	3275	1663	1552	1279	649	—	—
[Co(N_4_O_4_MacL_1_)Cl_2_]	3270	1689	1565	1277	648	429	322
[Co(N_4_O_4_MacL_2_)Cl_2_]	3259	1687	1581	1299	660	447	331
[Co(N_4_O_4_MacL_3_)Cl_2_]	3254	1679	1580	1309	661	455	343

### 3.3 ^1^H and ^13^C-NMR spectra

Proton NMR spectra provided additional confirmation for the successful synthesis of the amide-containing Macrocyclic ligands (N_4_O_4_MacL_1_–N_4_O_4_MacL_3_).^41^ The spectra, recorded in DMSO-*d*_6_ at room temperature (Fig. 3), indicate the stability of the ligand framework. The absence of signals corresponding to the primary amino protons of 1,8-diaminonaphthalene in the ^1^H NMR spectra suggests that the expected Macrocyclic structures were successfully formed. The presence of a broad peak between *δ* 9.76–10.11 ppm, assignable to amide –NH resonances, confirms the formation of amide linkages through condensation of amine groups with carboxyl moieties.^42^

**Fig. 2:**
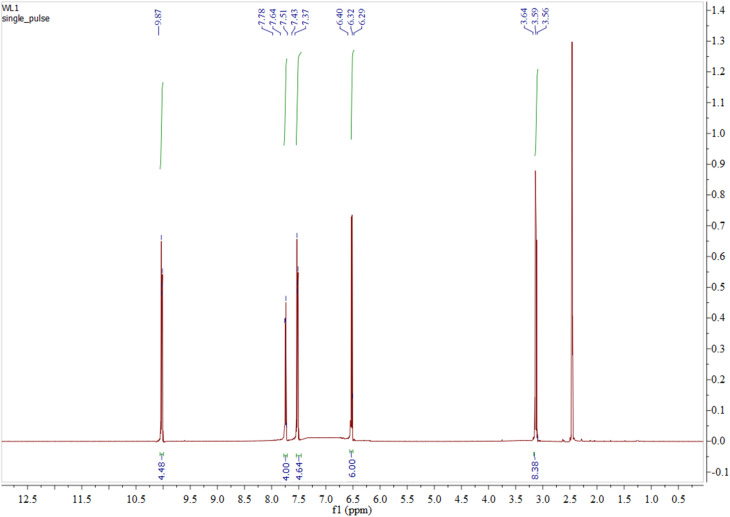
^1^H-NMR spectrum of tetra amide macrocyclic ligand N_4_O_4_MacL_1_.

**Fig. 3:**
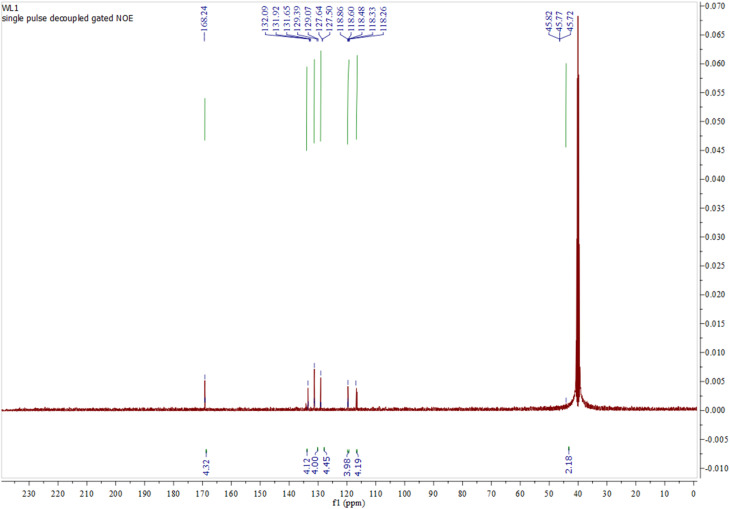
^13^C-NMR spectrum of tetra amide macrocyclic ligand N_4_O_4_MacL_1_.

The singlet signals at *δ* 3.75, 3.12, and 3.39 ppm in N_4_O_4_MacL_1_, N_4_O_4_MacL_2_, and N_4_O_4_MacL_3_, respectively, are attributed to the methylene protons (–CH_2_–) of the condensed diacid units (adipic, succinic, and glutaric acids). Additionally, slightly broad signals for amine protons appear between *δ* 6.13–6.52 ppm, while aromatic protons resonate in the *δ* 7.23–7.79 ppm range (Fig. 2).^43^ Characteristic aromatic proton resonances are also present, as detailed in Table 2. Further validation of Macrocycle formation and amide linkage incorporation is furnished by the ^13^C NMR spectral data. Broad peaks were observed at *δ* 165.36–168.85 ppm for amide carbons, *δ* 116.05–132.54 ppm for aromatic carbons, and *δ* 45.57–49.72 ppm for aliphatic carbons (Table 3 and Fig. 3),^44^ confirming the expected chemical environment of the macrocyclic frameworks.

**Table 2:** Proton NMR chemical shift assignments (ppm) for macrocyclic ligands (in ppm)

Ligands	(CO–NH) (m)	1,4-C_6_H_4_ (m)	1,2-C_6_H_4_ (m)	–CH_2_ (m)
[N_4_O_4_MacL_1_]	9.76	7.35–7.75	6.30–6.41	3.75–3.95
[N_4_O_4_MacL_2_]	9.89	7.23–7.79	6.22–6.52	3.12–3.35
[N_4_O_4_MacL_3_]	10.11	7.38–7.57	6.13–6.38	3.39–3.49

**Table 3:** Assigned ^13^C NMR resonances (ppm) for the amide-based macrocyclic ligands (in ppm)

Compound	(CO–NH)	1,4-C_6_H_4_	1,2-C_6_H_4_	–CH_2_ (m)
[N_4_O_4_MacL_1_]	167.13	116.05, 116.74	127.36, 132.26	45.57
[N_4_O_4_MacL_2_]	165.36	116.25, 117.08	127.32, 132.54	47.53
[N_4_O_4_MacL_3_]	168.85	116.87, 119.58	130.41, 132.15	49.72

### 3.4 Mass spectral

The ligands and their Co(ii) complexes were characterized by electrospray ionization mass spectrometry (ESI-MS) in DMSO solution. Confirmation of the ligand's molecular formula was obtained from its positive-ion ESI mass spectrum, which displayed a prominent molecular ion peak at *m*/*z* 480, corresponding to the molecular weight of C_28_H_24_ (calculated *m*/*z* 380.3413). The spectra of the ligands (N_4_O_4_MacL_1_–N_4_O_4_MacL_3_) and the divalent cobalt complexes exhibited prominent molecular ion peaks represented as M^*+*^ or (M + H)^+^.

In addition to the molecular ion peaks, the spectra displayed fragments resulting from the loss of phenyl or benzyl substituents. Several other characteristic peaks were also observed in the mass spectra, confirming the composition of the compounds. For example, the fragmentation pattern of N_4_O_4_MacL_2_ showed a molecular ion peak at *m*/*z* 480 (C_26_H_20_). The ligands N_4_O_4_MacL_1_, N_4_O_4_MacL_3_ and the corresponding Co(ii) complexes ([Co(N_4_O_4_MacL_1_)Cl_2_]–[Co(N_4_O_4_MacL_3_)Cl_2_]) exhibited molecular ion peaks at *m*/*z* 452.47, 508.1245, 580.4786, 608.6136, and 638.6136, respectively (Fig. S4–S9), supporting the proposed structures.

### 3.5 Electronic transitions and UV-vis spectral features

Magnetic moment measurements of metal ions are a valuable tool for determining electronic configurations and predicting molecular geometry.^47^ For the Co(ii) complexes, the observed magnetic moment of 4.84 B.M. corresponds to three unpaired electrons, consistent with a spin-only distorted octahedral geometry. Magnetic susceptibility of the synthesized complexes was measured using the Gouy balance method, employing a calibrated Gouy tube and using Hg[Co(SCN)_4_] as the standard reference material. Measurements were carried out at room temperature, and the necessary diamagnetic corrections for both the ligand framework and metal ions were applied using Pascal's constants. The corrected molar susceptibility values were then used to calculate the effective magnetic moment (µ_eff) according to the standard equation µ_eff = 2.828(*χ M T*)^1/2^.

The electronic spectra of the amide-containing macrocyclic ligands (N_4_O_4_MacL_1_–N_4_O_4_MacL_3_) display two prominent absorption bands in the ranges of 312–317 nm and 337–355 nm. The first band is attributed to the π → π* transitions within the amide chromophores,^45^ while the second band around 350 nm arises from *n* → π* transitions, superimposed on a charge-transfer process from the aromatic ring system to the amide group. Upon coordination with the metal center *via* the amide nitrogen atoms, coinciding with an electronic charge-transfer process originating from the aromatic ring and directed toward the amide functional group.

The electronic spectra and magnetic moment data of all Co(ii) complexes recorded at room temperature are consistent with the proposed distorted octahedral geometry (Table 4). Specifically, the Co(ii) complexes exhibit three d–d transition bands at 759–789 nm, 569–589 nm, and 439–461 nm, corresponding to the ^4^T_1_g → ^4^T_2_g (F), ^4^T_1_g → ^4^A_2_g, and ^4^T_1_g → ^4^T_2_g (P) transitions, respectively.^46^

**Table 4:** Electronic absorption data and magnetic susceptibility measurements for the macrocyclic ligands and their Co(ii) complexes

Compound	*M* (ohm^−1^ cm^2^ mol^−1^	*λ*_max_ (nm)
[N_4_O_4_MacL_1_]	—	312, 359
[N_4_O_4_MacL_2_]	—	316, 341
[N_4_O_4_MacL_3_]	—	319, 349
[Co(N_4_O_4_MacL_1_)Cl_2_]	14.8	449, 589, 765
[Co(N_4_O_4_MacL_2_)Cl_2_]	15.9	439, 590, 795
[Co(N_4_O_4_MacL_3_)Cl_2_]	13.7	461, 580, 782

### 3.6 EPR

EPR spectroscopy was employed to investigate the presence of the paramagnetic electrons associated with the Co(ii) coordination centers. The spectra of [Co(N_4_O_4_MacL_1_)Cl_2_]–[Co(N_4_O_4_MacL_3_)Cl_2_] were recorded in the solid state at liquid nitrogen temperature (77 K) and as polycrystalline samples dissolved in acetonitrile. Comparable *g*-values in solid-state and solution measurements confirm the presence of one unpaired electron in the d_*x*^2^−*y*^2^_ orbital. This observation aligns with a distorted octahedral stereochemistry for the Co(ii) complexes.^48^

The EPR data (Table S2) exhibited the characteristic relation *g*_∥_ > *g*_⊥_ > 2, consistent with axial symmetry. From these spectra, both *g*-values and the spin–orbit coupling parameter *G* were calculated. The shift relative to the free-electron *g*-value (2.0023) reflects appreciable orbital angular momentum involvement, corroborating the distorted octahedral geometry proposed for the Co(ii) complexes.

### 3.7 Thermogravimetric analysis

Thermal analysis is commonly used to evaluate the thermal stability of ligands (N_4_O_4_MacL_1_–N_4_O_4_MacL_3_) and their Co(ii) complexes ([Co(N_4_O_4_MacL_1_)Cl_2_]–[Co(N_4_O_4_MacL_3_)Cl_2_]) and to detect the presence of solvent molecules within the coordination sphere. Thermogravimetric analysis (TGA) was carried out for all synthesized compounds over the temperature range of 50–700 °C. The thermograms of the complexes exhibited similar patterns.

**Fig. 4:**
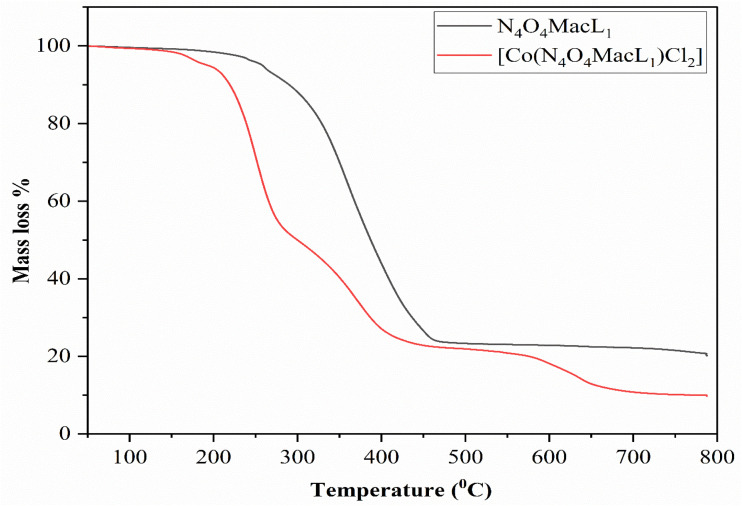
Thermogravimetric analysis data of (N_4_O_4_MacL_1_) and Co(ii) complex [Co(N_4_O_4_MacL_1_)Cl_2_].

Representative TGA curves of N_4_O_4_MacL_1_ and [Co(N_4_O_4_MacL_1_)Cl_2_] indicated no significant weight loss up to 160 °C, confirmed that no water molecules were present either inside or outside the coordination environment. The decomposition of [Co(N_4_O_4_MacL_1_)Cl_2_] occurred in three distinct stages. The first stage, between 230–430 °C, involved a mass loss of 19.39% (calcd. 20.08%), corresponding to the loss of two chloride ions. The second stage, from 160–210 °C, accounted for the decomposition of the organic ligand framework, resulting in a 44.35% mass loss (calcd. 46.78%).^49^ The third stage, observed between 570–640 °C, involved a further weight loss of 15.46% (calcd. 17.36%) due to the breakdown of the remaining coordinated ligand moiety. Above 640 °C, the TGA curve became nearly horizontal, indicating the formation of the final metal oxide (Fig. 4).

The experimentally observed mass losses for [Co(N_4_O_4_MacL_1_)Cl_2_] closely matched the calculated values based on its proposed chemical formula. These results demonstrate that the complexes are thermally stable up to 640 °C, and similar thermal behavior was observed for all other complexes.

#### 3.7.1 Kinetic parameters

The kinetic and thermodynamic behavior associated with the non-isothermal decomposition of the synthesized macrocyclic ligands (N_4_O_4_MacL_1_–N_4_O_4_MacL_3_) and their cobalt(ii) complexes was systematically investigated using thermogravimetric data. Kinetic parameters were evaluated using the Ozawa-Flynn-Wall (OFW) isoconversional technique in conjunction with the Coats–Redfern integral model, enabling detailed insight into the thermal degradation mechanism and decomposition pathway.^50,51^ Key activation parameters, including activation energy (*E*_a_), pre-exponential factor (*Z*), reaction order (*n*), entropy of activation (Δ*S*), enthalpy of activation (Δ*H*), Gibbs free energy of activation (Δ*G*), and correlation coefficients (r), were calculated for each thermal event. The OFW model provided activation energies without assuming a specific reaction mechanism, while the Coats–Redfern procedure allowed mechanistic interpretation through comparison with established kinetic models. Thermodynamic activation functions were further computed using the Eyring transition-state equation,^52,53^ offering deeper insight into the energetic requirements and molecular rearrangements accompanying thermal degradation. Positive Δ*G* values indicated non-spontaneous decomposition processes, whereas the sign and magnitude of Δ*S*^‡^ suggested the extent of disorder associated with the transition state. Additionally, comparative analysis between free ligands and their Co(ii) complexes demonstrated enhanced thermal stability upon metal coordination, consistent with stronger chelate–metal interactions and increased lattice energy in the complexes. These comprehensive thermal studies contribute to understanding the stability profiles, degradation pathways, and molecular rigidity imparted by cobalt coordination, providing valuable information for their potential applications in catalysis, coordination chemistry, and materials science.1
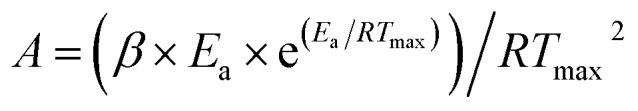
2
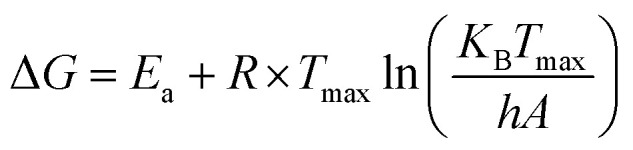

3 Δ*H* = *E*_a_ + *RT*_max_

4
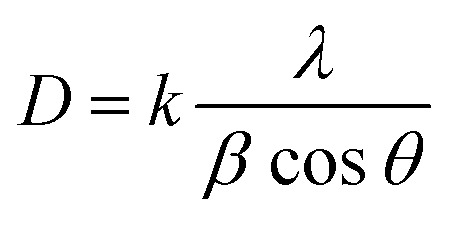


*h* = Planck constant (6.626 × 10^−34^ J s^−1^) and *K*_B_ = Boltzmann constant (1.38 10^−23^ J s^−1^).

**Fig. 5:**
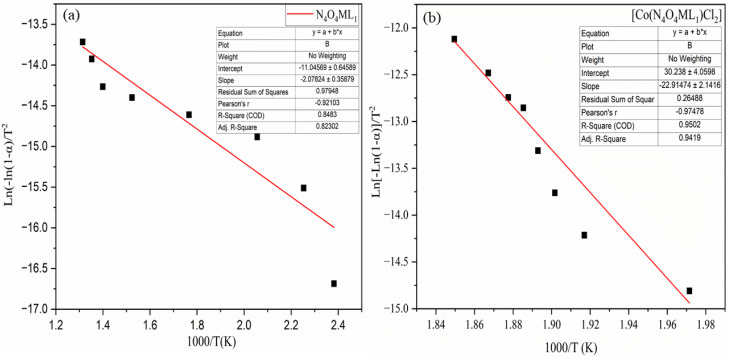
Coats–Redfern kinetic plots for macrocyclic ligand N_4_O_4_MacL_1_ and the corresponding Co(ii) complex [Co(N_4_O_4_MacL_1_)Cl_2_].

**Fig. 6:**
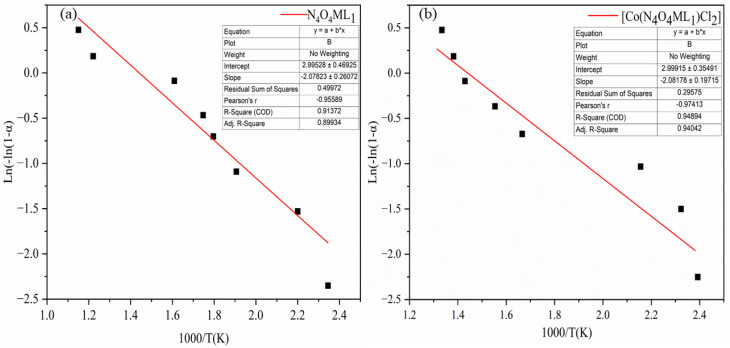
Ozawa fyn-wall kinetic plots for macrocyclic ligand N_4_O_4_MacL_1_ and the corresponding [Co(N_4_O_4_MacL_1_)Cl_2_].

The data are presented in Tables 5 and 6. The results indicate that the values obtained from the two analytical methods are in close agreement (Fig. 5 and 6, S8–S11). The relatively low activation energy values for the Co(ii) complexes suggest an autocatalytic effect of the metal ions on the thermal decomposition process. The observed negative entropy of activation (Δ*S*^‡^) values indicate a slow reaction, with the activated complex being more ordered than the initial reactants. This increased order in the transition state may be attributed to bond polarization, potentially facilitated by charge transfer electronic transitions during the decomposition process.

**Table 5:** Coats–Redfern kinetic data for macrocyclic ligands (N_4_O_4_MacL_1_–N_4_O_4_MacL_3_) and corresponding cobalt(ii) complexes

Compounds	*R*	(*T*_max_)	(*E*_a_)	*A*	Δ*G*	Δ*H*	Δ*S*
[N_4_O_4_MacL_1_]	0.85383	571.18	13.532	0.00145	174.788 ± 0.65	−4.115 ± 0.03	−294.25 ± 1.8
[N_4_O_4_MacL_2_]	0.82104	760.39	16.657	0.00129	220.897 ± 0.72	−6.225 ± 0.05	−281.25 ± 2.1
[N_4_O_4_MacL_3_]	0.93138	540.49	36.654	0.00445	158.044 ± 0.58	−4.248 ± 0.03	−285.19 ± 1.7
[Co(N_4_O_4_MacL_1_)Cl_2_]	0.94602	734.28	15.673	0.00029	238.025 ± 0.74	−6.654 ± 0.04	−313.45 ± 2.3
[Co(N_4_O_4_MacL_2_)Cl_2_]	0.91324	612.18	180.12	0.00689	172.843 ± 0.63	−4.514 ± 0.05	−274.58 ± 1.9
[Co(N_4_O_4_MacL_3_)Cl_2_]	0.94214	541.13	356.58	0.0399	167.435 ± 0.57	−4.320 ± 0.03	−301.45 ± 2.0

**Table 6:** Thermal data of (N_4_O_4_MacL_1_–N_4_O_4_MacL_3_) and [Co(N_4_O_4_MacL_1_)Cl_2_]–[Co(N_4_O_4_MacL_1_)Cl_2_] complexes by Ozawa-Fyn Wall method

Compound	*R*	*T*_max_	*E*_a_	*A*	Δ*G*	Δ*H*	Δ*S*
[N_4_O_4_MacL_1_]	0.91245	574.33	18.465	0.0024	175.252	−4.786	−291.72
[N_4_O_4_MacL_2_]	0.9487	761.32	257.24	0.0155	218.148	−6.205	−279.73
[N_4_O_4_MacL_3_]	0.85845	544.42	21.520	0.0023	162.684	−4.678	−291.43
[Co(N_4_O_4_MacL_1_)Cl_2_]	0.93745	727.73	27.450	0.0019	225.402	−6.754	−294.82
[Co(N_4_O_4_MacL_2_)Cl_2_]	0.91248	612.92	46.536	0.0042	189.121	−5.221	−288.08
[Co(N_4_O_4_MacL_3_)Cl_2_]	0.95471	537.09	250.25	0.0344	144.765	−4.188	−265.74

### 3.8 PXRD analysis

**Fig. 7:**
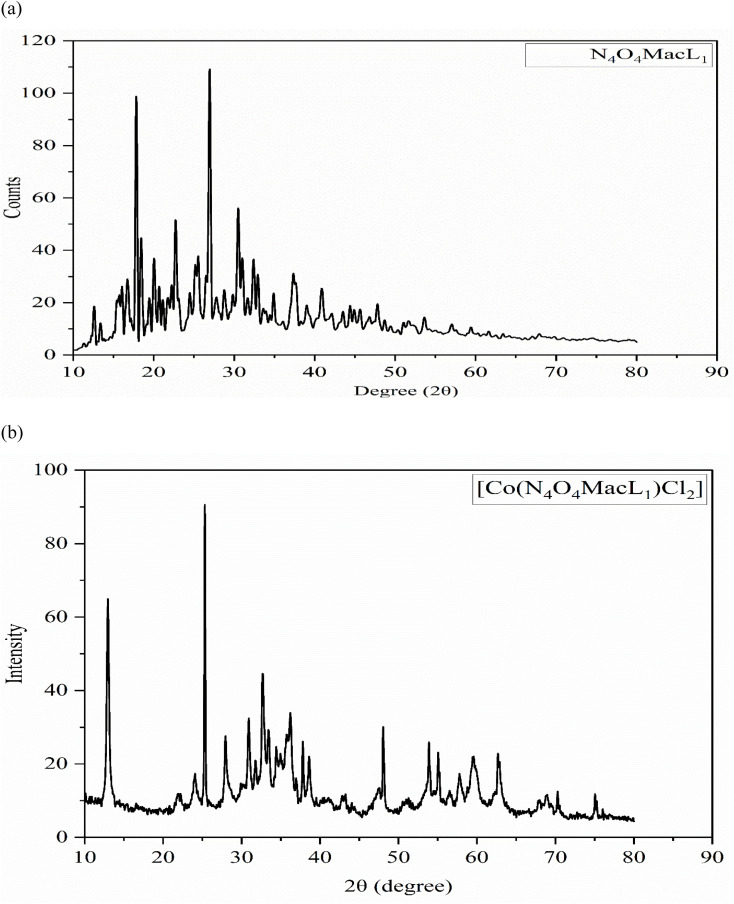
Powder X-ray diffraction profiles of (a) macrocyclic ligand N_4_O_4_MacL_1_ and (b) the corresponding cobalt(ii) complex [Co(N_4_O_4_MacL_1_)Cl_2_].

**Fig. 8:**
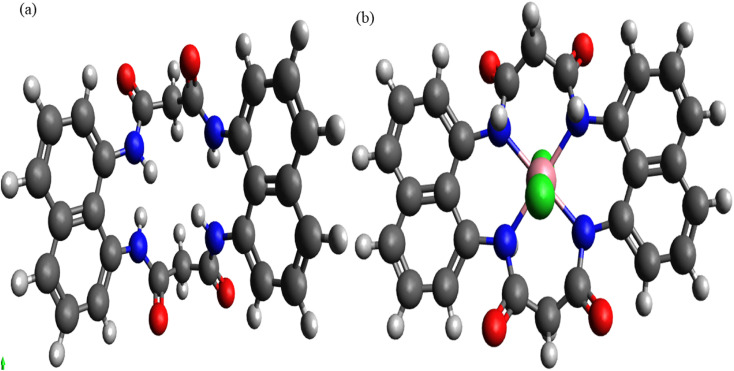
DFT-optimized molecular geometries of the macrocyclic ligand N_4_O_4_MacL_1_ and the corresponding Co(ii) complex [Co(N_4_O_4_MacL_1_)Cl_2_].

The powder X-ray diffraction (XRD) patterns of the ligands (N_4_O_4_MacL_1_–N_4_O_4_MacL_3_) and their Co(ii) complexes ([Co(N_4_O_4_MacL_1_)Cl_2_]–[Co(N_4_O_4_MacL_3_)Cl_2_]) are shown in Fig. 8. Multiple diffraction peaks indicate the polycrystalline nature of the samples. Crystals suitable for single-crystal XRD analysis could not be obtained for the Co(ii) complexes. Therefore, the powder XRD data were used to investigate the crystal structures. The ligand [N_4_O_4_MacL_1_] (Fig. 7a) exhibited sharp, well-defined peaks, confirming its crystalline nature, whereas the complex [Co(N_4_O_4_MacL_1_)Cl_2_] (Fig. 8b) showed broader and less intense peaks, indicative of partial amorphous character. A close correlation was observed between the theoretical and experimental *d*-spacing values for the complexes. Crystallite dimensions were determined using the Debye–Scherrer formula (eqn (1)), employing the diffraction line of maximum intensity for the calculation.^54^
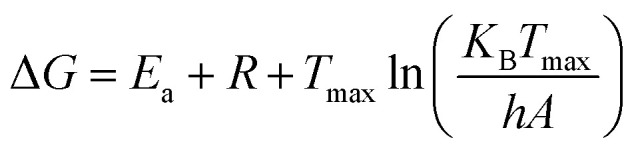


The crystallite size (*D*) was calculated using the Debye–Scherrer equation, where *k* is the shape factor (0.94 for Cu Kα radiation), *λ* is the X-ray wavelength (1.5406 Å), *θ* is the Bragg diffraction angle, and *β* is the full width at half maximum (FWHM) of the diffraction peak. Using this approach, the crystallite size of the complex [Co(N_4_O_4_MacL_1_)Cl_2_] was determined to be 44.34 nm. Structural analysis indicates that [Co(N_4_O_4_MacL_1_)Cl_2_] adopts an orthorhombic crystal system. According to the result of powder X-ray diffraction studies, it has been found that the crystal system of macrocyclic complex [Co(N_4_O_4_MacL_1_)Cl_2_] orthorhombic with the unit cell parameters of *a* = 7.758, *b* = 17.542, *c* = 5.652 and *α* = *β* = *γ* = 90°, respectively. Table 7 reports the 2*θ* angles and Miller indices *h*, *k*, and *l* for each *d* value.

**Table 7:** Powder X-ray diffraction data for the macrocyclic complex [Co(N_4_O_4_MacL_1_)Cl_2_]

Peak no.	2*θ* (obs.)	2*θ* (calc.)	*h*	*k*	*l*	*d*-spacing (obs.)	*d*-spacing (calc.)
1	10.241	10.238	0	2	0	8.847	8.838
2	11.845	11.798	1	1	0	7.265	7.254
3	14.312	14.307	1	2	0	6.012	6.025
4	15.412	15.405	0	1	1	5.545	5.565
5	18.549	18.534	1	3	0	4.354	4.348
6	20.542	20.525	1	1	1	4.615	4.605
7	21.845	21.868	1	2	1	4.021	4.034
8	22.372	22.358	0	3	1	4.451	4.449
9	23.145	23.130	2	0	0	3.393	3.387
10	23.469	23.472	2	1	0	3.215	3.224
11	23.278	23.225	1	4	0	3.054	3.016
12	24.255	24.241	1	3	1	3.015	3.025
13	24.742	24.725	2	2	0	3.152	3.139
14	26.263	26.235	2	3	0	3.012	3.042
15	27.742	27.768	2	0	1	3.651	3.645
16	28.125	28.104	2	1	1	3.178	3.164
17	28.654	28.635	1	4	1	3.214	3.205
18	30.158	30.145	2	2	1	3.135	3.115

### 3.9 Molecular modelling

The molecular structures of the ligands (N_4_O_4_MacL_1_–N_4_O_4_MacL_3_) and their Co(ii) complexes were modelled using the ORCA program in combination with Avogadro 4.0 software.^55^ Molecular mechanics methods were first applied to pre-optimize the regions of the molecules not directly coordinated to the metal centre. Energy minimization was performed iteratively for each molecule until convergence was achieved. In the optimized structures, the ligands adopt a highly folded conformation (Fig. 8), with the four oxygen atoms alternately displaced from the coordination plane constituted by the nitrogen atoms.

**Fig. 9:**
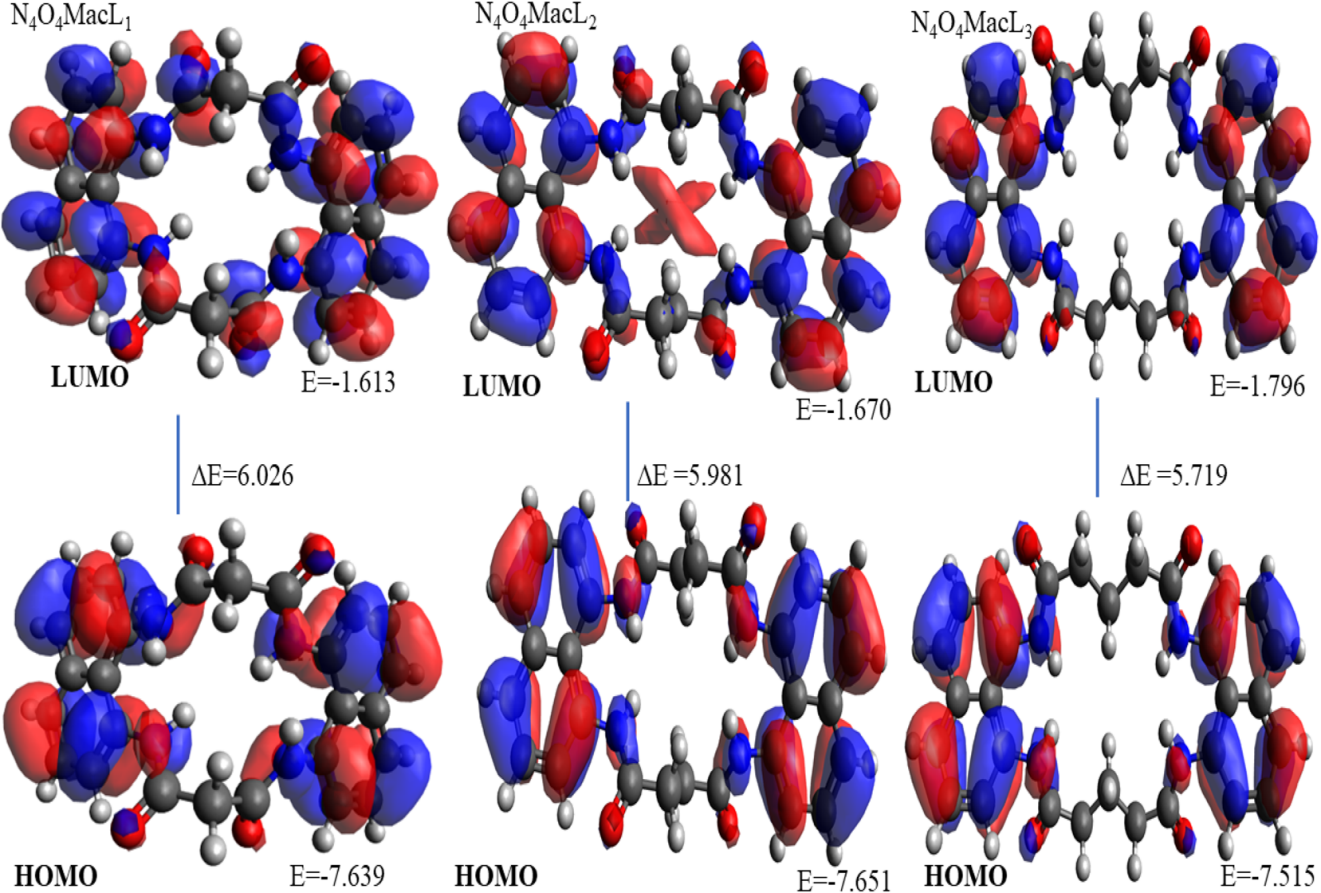
3D plots illustrating the HOMO and LUMO energy distributions for ligands N_4_O_4_MacL_1_–N_4_O_4_MacL_3_.

For the [Co(N_4_O_4_MacL_1_)Cl_2_] complex, the four nitrogen donor atoms occupy the equatorial plane, creating a distorted octahedral geometry around the Co(ii) centre (Fig. 9). The Co(ii) ion is situated near the mean plane of the equatorial donor set. Comparison of bond lengths (Å) and bond angles (°) for the ligands and complexes (Tables 8 and 9) reveals variations that reflect the coordination interactions between the ligand donor atoms and the metal ion.^53,54^ The equatorial Co–N distances are 2.09, 2.29, and 2.05 Å, while the two axial Co–Cl bonds are measured at 2.298–2.299 Å. These values are consistent with those reported in the literature.^56^

**Table 8:** DFT-derived bond length values (Å) for macrocyclic ligands and complexes [Co(N_4_O_4_MacL_1_)Cl_2_–Co(N_4_O_4_MacL_3_)Cl_2_]

Compounds	(C=O)	(C–N)	(N–H)	(Co–N)	(Co–Cl)
[N_4_O_4_MacL_1_]	1.21232	1.35514	1.02512	—	—
[N_4_O_4_MacL_2_]	1.21295	1.36214	1.02114	—	—
[N_4_O_4_MacL_3_]	1.22104	1.36254	1.02385	—	—
[Co(N_4_O_4_MacL_1_)Cl_2_]	1.22363	1.44125	1.02745	2.11325	2.28979
[Co(N_4_O_4_MacL_2_)Cl_2_]	1.22145	1.45412	1.03954	2.35214	2.25347
[Co(N_4_O_4_MacL_3_)Cl_2_]	1.24125	1.43895	1.03745	2.06578	2.27412

**Table 9:** The DFT-optimized bond angle values (°) corresponding to the complex [Co(N_4_O_4_MacL_3_)Cl_2_]

Atom-to-atom linkage	(Bond angles (°)	(Atomic connectivity)	(Bond angles (°)
N–Co–N	89.2854	N–Co–Cl	88.9874
N–Co–N	88.1845	N–Co–Cl	90.4512
N–Co–N	178.1245	N–Co–Cl	91.0215
N–Co–Cl	86.4578	N–Co–Cl	83.1245
N–Co–Cl	93.5641	Cl–Co–Cl	176.4512
N–Co–N	178.3254		
N–Co–N	91.1245		
N–Co–Cl	88.5741		
N–Co–Cl	91.0215		
N–Co–N	91.0145		

#### 3.9.1 Evaluation of global quantum chemical descriptors

The structural configurations of the amide-linked macrocyclic systems and their Co(ii) derivatives were optimized using density functional theory (DFT),^57,58^ and the resulting structures are depicted in Fig. 9.^59^ The calculated bond lengths and bond angles for the ligands and complexes are summarized in Tables 8 and 9.

Frontier molecular orbitals, specifically the highest occupied molecular orbital (HOMO) and the lowest unoccupied molecular orbital (LUMO),^60^ play a key role in determining chemical stability. The HOMO represents the electron-donating ability of the molecule, while the LUMO corresponds to its electron-accepting capability. In addition, several global reactivity descriptors were calculated, including the frontier molecular orbital energy gap (Δ*E*) between HOMO and LUMO levels, the global descriptors *χ*, *µ*, *η*, *S*, and *ω* representing electronegativity, chemical potential, hardness, softness, and electrophilicity, respectively, and the maximum additional electronic charge (Δ*N*_max), with values reported in Table 10.

**Table 10:** Global parameters of ligands (ML_1_–ML_3_) and complexes [Co(N_4_O_4_MacL_1_)Cl_2_–Co(N_4_O_4_MacL_3_)Cl_2_]

Parameters	(N_4_O_4_MacL_1_)	(N_4_O_4_MacL_2_)	(N_4_O_4_MacL_3_)	9(*a*)	9(*b*)	9(*c*)
*E*_HOMO_ (eV)	−7.358	−7.547	−7.145	−6.754	−6.124	6.245
*E*_LUMO_ (eV)	−1.541	−1.812	−1.325	−2.214	−2.147	−2.149
Δ*E* (eV)	6.124	5.504	5.0154	4.387	3.0215	4.213
IE (eV)	7.541	7.648	7.745	6.145	6.358	6.345
*χ* (eV)	4.314	4.4123	4.2154	4.845	4.6587	4.4045
*η* (eV)	3.145	2.8745	2.7945	2.124	1.7458	2.1254
*S* (eV)^−1^	0.1287	0.1145	0.0158	0.2154	0.2584	0.2545
*ω* (eV)	3.5412	3.7452	4.048	5.5845	5.1248	4.2541
Pi	−4.625	−4.2514	−4.6145	−4.336	−4.5142	−4.1478
*N*_max_	1.4875	1.2014	1.2548	2.0987	2.0215	2.32514

The HOMO–LUMO energy gap (Δ*E* = 4.49 eV) is an important parameter reflecting molecular stability; higher values indicate greater stability. The negative chemical potential (*µ*) combined with a positive electrophilicity index (*ω*) suggests that the amide-based ligands are capable of donating electrons to metal centers, supporting their role in complex formation.

### 3.10 Biological activities

#### 3.10.1 Antimicrobial bustle

**Fig. 10:**
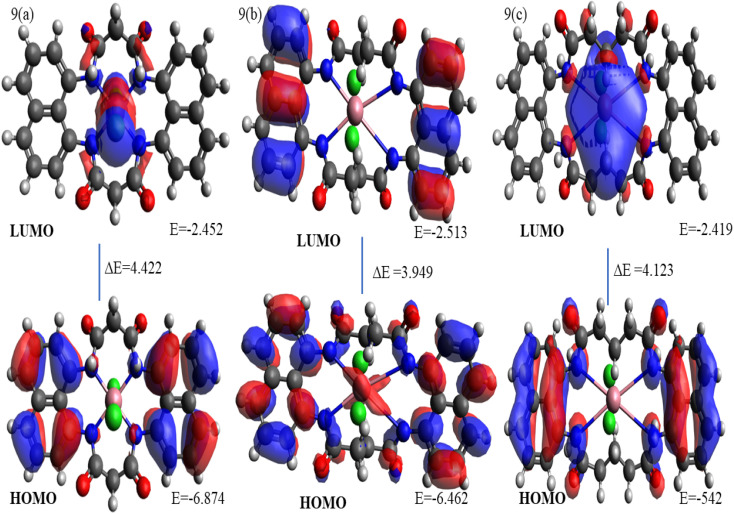
3D plots illustrating the HOMO and LUMO energy distributions for the Co(ii) complexes.

The amide-containing macrocyclic ligands (N_4_O_4_MacL_1_–N_4_O_4_MacL_3_), featuring nitrogen and oxygen donor atoms, represent an important class of molecules with significant medicinal applications. These ligands exhibit notable biological activities, including antibacterial^61,62^ and anticancer effects. The ligands and their corresponding Co(ii) complexes were assessed for antibacterial efficacy against representative Gram-positive (*S. aureus*, *B. subtilis*) and Gram-negative (*E. coli*, *S. typhi*) bacterial pathogens. Ampicillin served as the standard reference drug, and DMSO was used as the solvent. The compounds were first dissolved in a small volume of DMSO/methanol (ensuring complete dissolution) and then diluted with the culture medium, keeping the final solvent concentration below 1% v/v to avoid any solvent-induced antimicrobial effect. Corresponding solvent controls were also included and showed no inhibitory activity. The bactericidal results of the synthesized compounds are summarized in Table 11, while Fig. 12 illustrates their activity against the four bacterial strains (Fig. 10).

**Table 11:** Comparative antibacterial screening of Macrocyclic ligands and cobalt(ii) complexes *via* agar diffusion, presented as inhibition zone sizes (mm) and % of reference drug

Compounds	Inhibition zone (mm)							
	*B. subtilis*		*S. aureus*		*E. coli*		*S. Typhi*	
Conc. (µg ml^−1^)	30.0	50	30.0	50.0	30.0	50.0	30.0	50.0
[N_4_O_4_MacL_1_]	8	9	6	8	10	11	7	9
[N_4_O_4_MacL_2_]	10	11	8	9	12	13	10	10
[N_4_O_4_MacL_3_]	9	10	7	8	13	17	9	11
[Co(N_4_O_4_MacL_1_)Cl_2_]	10	11	9	10	19	20	13	14
[Co(N_4_O_4_MacL_2_)Cl_2_]	12	14	10	11	18	21	15	16
[Co(N_4_O_4_MacL_3_)Cl_2_]	14	17	9	10	17	19	14	17
Ampicillin	16	20	11	13	24	26	18	20

**Fig. 11:**
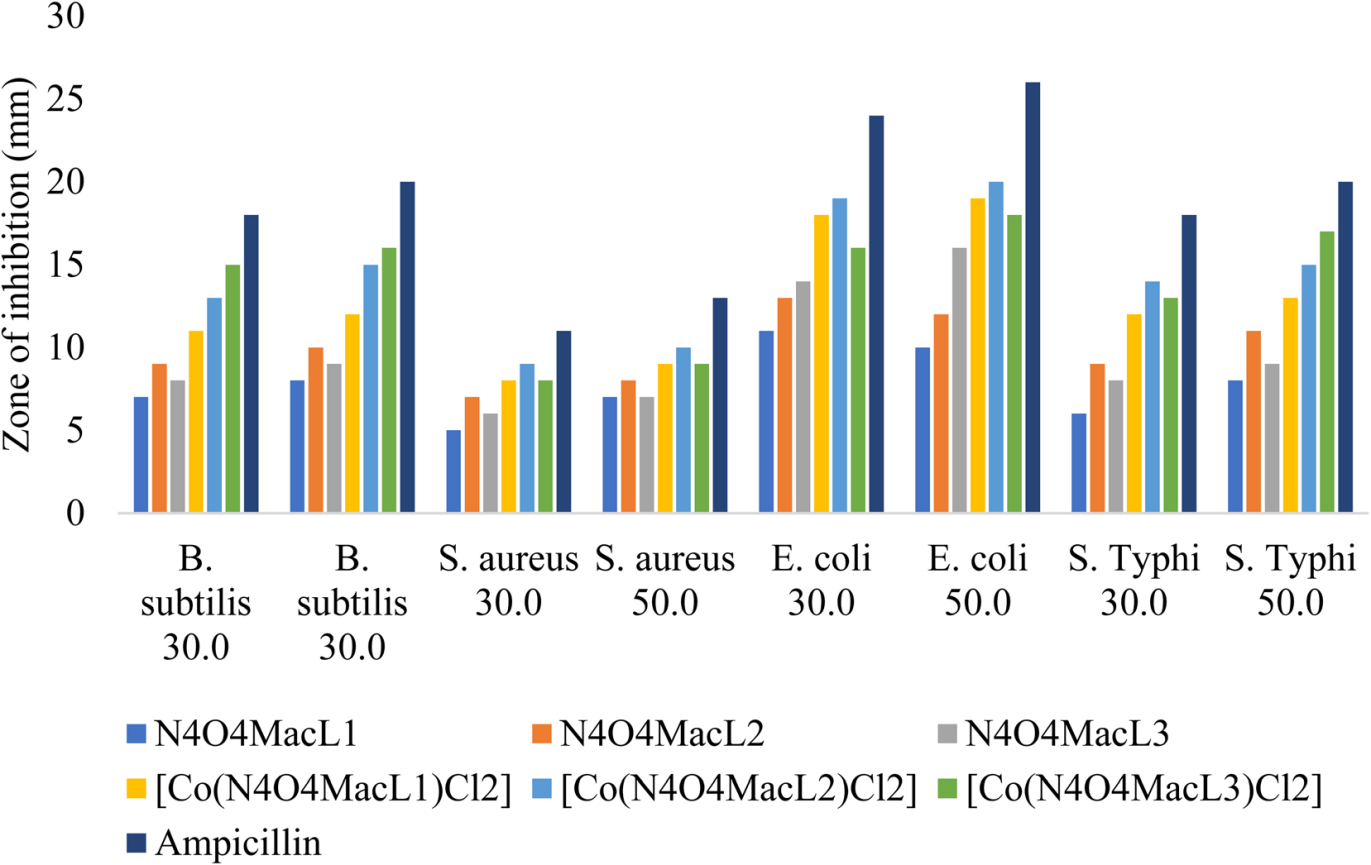
Zone of bacterial growth inhibition (mm) exhibited by the synthesized compounds.

The enhanced antibacterial activity of the Co(ii) complexes can be rationalized using Tweedy's chelation theory and Overtone's concept of cell permeability. Chelation reduces the polarity of the central metal ion due to partial sharing of its positive charge with the ligand donor atoms, which in turn increases the lipophilicity of the complex. Increased lipophilicity facilitates the penetration of the complex through the lipid bilayer of bacterial cell membranes, consistent with Overtone's idea that only lipid-soluble molecules efficiently cross cell membranes. Enhanced electron delocalization within the chelate ring further supports membrane permeability. Once inside the cell, the metal complexes can interact with bacterial enzymes, potentially inhibiting key biochemical pathways such as respiration, ultimately leading to cell death.^63–65^ The relative antibacterial potency of the complexes follows the order: [Co(N_4_O_4_MacL_2_)Cl_2_] > [Co(N_4_O_4_MacL_3_)Cl_2_] > [Co(N_4_O_4_MacL_1_)Cl_2_] (Fig. 11).

**Fig. 12:**
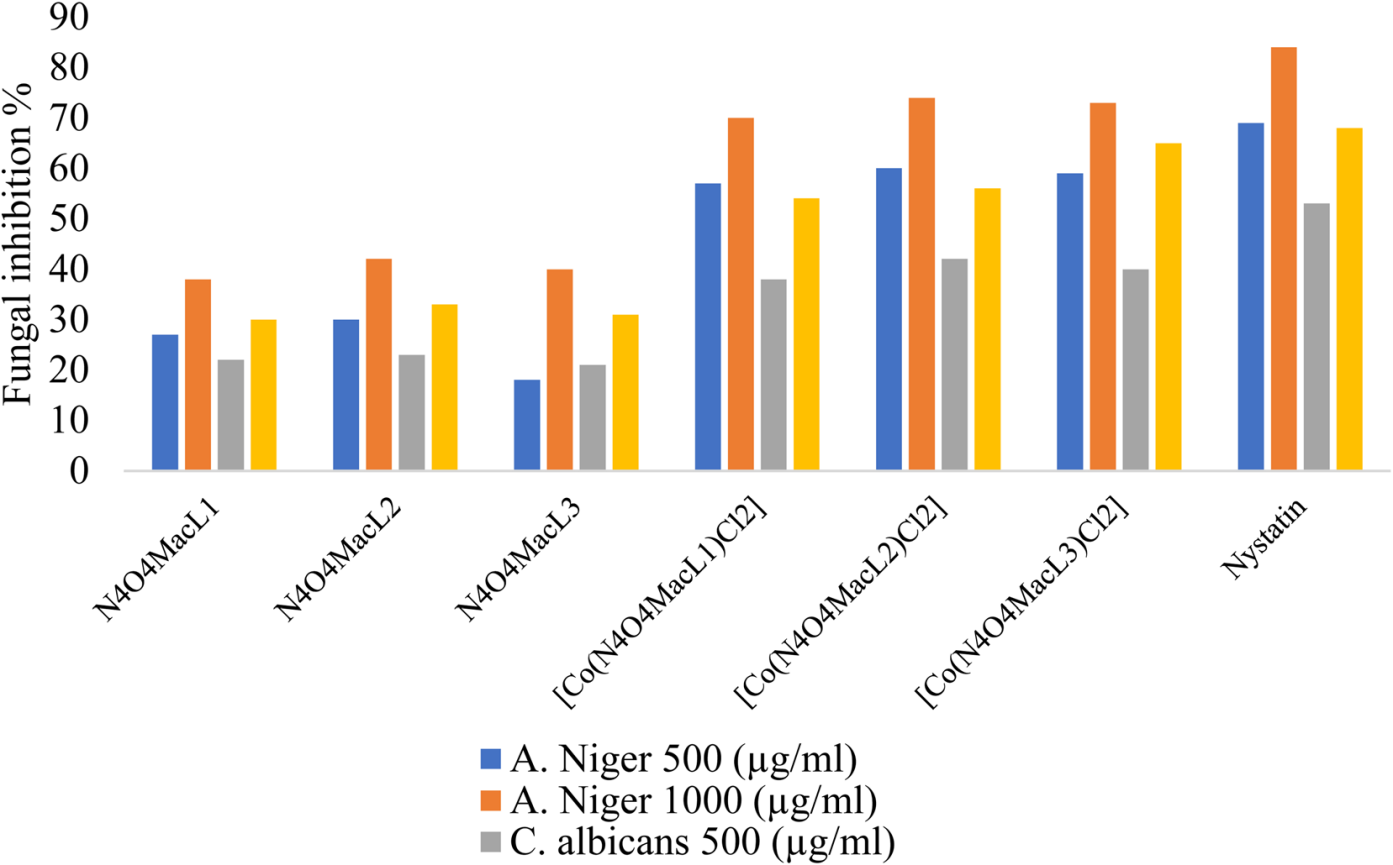
*In vitro* antifungal activity of the compounds expressed as inhibition zone diameters (mm).

The antifungal activity of the compounds was evaluated *in vitro* against *Aspergillus niger* and *Candida albicans* using the serial dilution method. The minimum inhibitory concentration (MIC) values are listed in Table 12. Comparison of the MIC data indicates that the Co(ii) complexes display greater antifungal activity than the free ligands. Among the tested compounds, [Co(N_4_O_4_MacL_2_)Cl_2_] exhibited the highest antifungal efficacy relative to the other complexes and the standard antifungal agent (Fig. 12).

**Table 12:** Antimicrobial MIC (µM) of ligands and Co(ii) complexes

Compounds	Fungal inhibition % (conc. in µg ml^−1^)			
	*A. Niger*		*C. albicans*	
Conc. (µg ml^−1^)	500 (µg ml^−1^)	1000 (µg ml^−1^)	500 (µg ml^−1^)	1000 (µg ml^−1^)
[N_4_O_4_MacL_1_]	28	39	23	31
[N_4_O_4_MacL_2_]	31	43	24	32
[N_4_O_4_MacL_3_]	19	41	22	32
[Co(N_4_O_4_MacL_1_)Cl_2_]	58	71	39	55
[Co(N_4_O_4_MacL_2_)Cl_2_]	61	75	43	57
[Co(N_4_O_4_MacL_3_)Cl_2_]	60	74	41	66
Nystatin	69	84	53	68

#### 3.10.2 Antioxidant activity

**Fig. 13:**
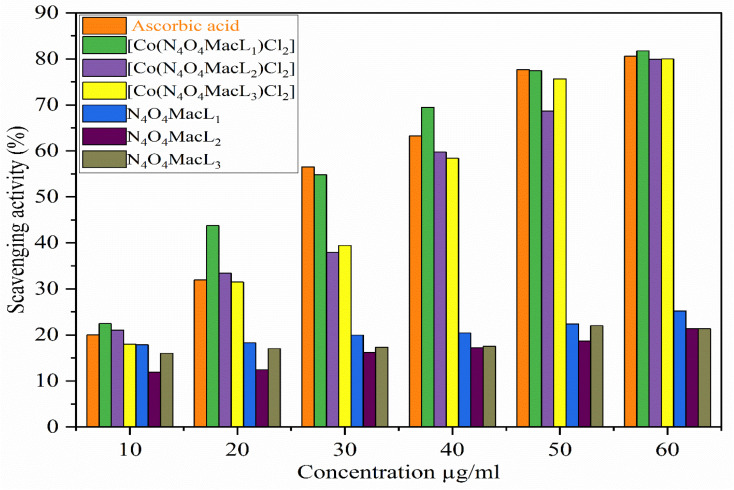
DPPH radical scavenging activity of the synthesized ligands and their Co(ii) complexes.

**Fig. 14:**
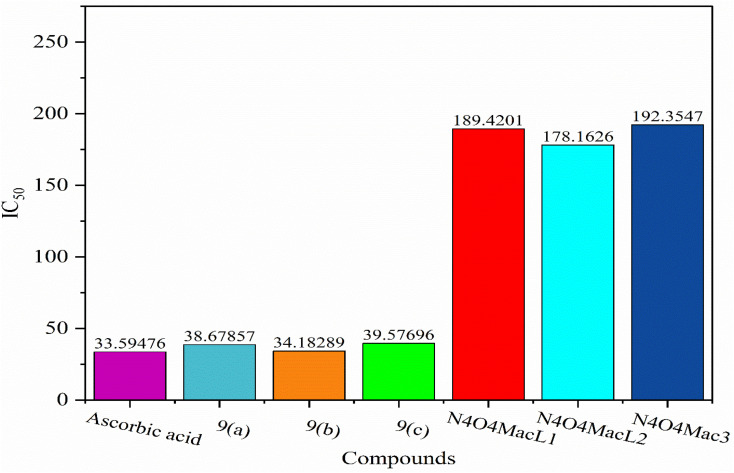
Evaluation of free-radical scavenging activity for ligands (N4O4MacL_1_–N4O4MacL_3_) and complexes.

The synthesized ligands (N_4_O_4_MacL_1_–N_4_O_4_MacL_3_) and their Co(ii) complexes ([Co(N_4_O_4_MacL_1_)Cl_2_]–[Co(N_4_O_4_MacL_3_)Cl_2_]) were evaluated for their antioxidant activity by examining their ability to scavenge DPPH radicals. The results indicate that the Co(ii) Macrocyclic complexes exhibit significantly higher antioxidant activity compared to the free ligands. DPPH, which possesses an unpaired electron, shows a strong absorption band at 517 nm; upon accepting a hydrogen atom or electron from the test compounds, it is reduced to a more stable diamagnetic form, resulting in decreased absorbance.^66^Fig. 13 and 14 illustrate the radical scavenging activity of the compounds at various concentrations. The antioxidant activity of the amide-based ligands is largely attributed to their H-donating ability, which allows deprotonation and stabilization of the resulting radical. Radical-scavenging performance is largely governed by the dissociation behavior of the carboxylic hydroxyl groups within the ligand framework. All measurements were performed in duplicate and averaged for accuracy. When compared to the standard antioxidant, ascorbic acid, the Co(ii) complexes demonstrated excellent radical-scavenging capability. This enhanced activity is likely due to the ability of the metal complexes to transfer electrons from the HOMO to the DPPH radical, stabilizing it and lowering the absorption maximum.^67,68^

#### 3.10.3 Cytotoxicity

**Fig. 15:**
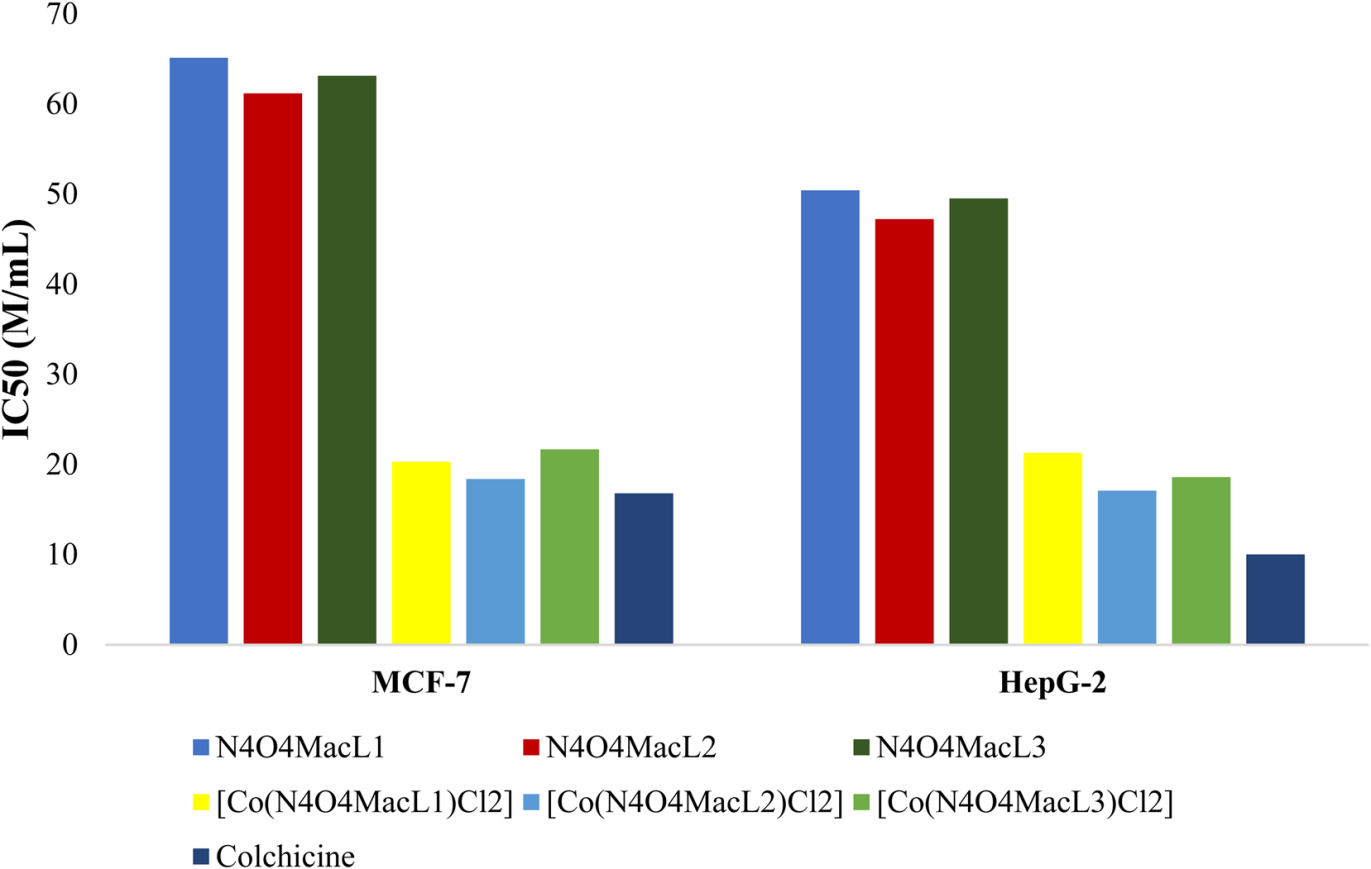
Comparative cytotoxic evaluation of macrocyclic ligands (N_4_O_4_MacL_1_–N_4_O_4_MacL_3_) and the corresponding Co(ii) complexes.

*In vitro* cytotoxic assessments were conducted on the prepared macrocyclic ligands and their corresponding Co(ii) complexes to investigate their anticancer potential against MCF-7 and HepG-2 cell lines at concentrations of 3.125, 6.250, 12.500, 25.000, 50.000, and 100.000 µM mL^−1^ using the MTT assay. The cancer cell line employed in this investigation was kindly provided by a laboratory at the Indian Institute of Science (IISc), Bengaluru, India, and was maintained under standard culture conditions. The results (Fig. 15) indicate that the Co(ii) complexes exhibit significantly higher cytotoxicity compared to the free ligands. Based on the calculated IC_50_ values, the Co(ii) complexes showed potent activity against MCF-7 cells while having minimal toxicity toward normal cells. Table 13 summarizes the *in vitro* cytotoxicity of the ligands (N_4_O_4_MacL_1_–N_4_O_4_MacL_3_), their Co(ii) complexes ([Co(N_4_O_4_MacL_1_)Cl_2_]–[Co(N_4_O_4_MacL_3_)Cl_2_]), and the standard drug colchicine. Colorimetric cytotoxicity assays revealed that the ligands displayed moderate activity against MCF-7 cells, with IC_50_ values ranging from 6.19–64.12 µM, whereas the Co(ii) complexes exhibited significantly enhanced cytotoxicity. Against HepG-2 cells, the ligands showed only marginal activity, while the Co(ii) complexes demonstrated strong cytotoxic effects with IC_50_ values between 47.23–50.42 µM. Among the ligands, N_4_O_4_MacL_1_ exhibited the lowest cytotoxic potential. These results highlight that cytotoxicity is largely influenced by the presence of the metal center and its interaction with the cancer cell lines.

**Table 13:** Half-maximal inhibitory concentration (IC_50_, µM) values for tetraamide macrocyclic ligands and corresponding cobalt(ii) complexes

Compounds	MCF-7	HepG-2
[N_4_O_4_MacL_1_]	65.01 ± 0.19	50.42 ± 0.66
[N_4_O_4_MacL_2_]	61.20 ± 0.18	47.23 ± 0.57
[N_4_O_4_MacL_3_]	63.11 ± 0.21	49.41 ± 0.61
[Co(N_4_O_4_MacL_1_)Cl_2_]	20.13 ± 0.31	21.38 ± 0.13
[Co(N_4_O_4_MacL_2_)Cl_2_]	18.42 ± 0.25	17.94 ± 0.45
[Co(N_4_O_4_MacL_3_)Cl_2_]	21.71 ± 0.45	18.62 ± 0.49
Colchicine	16.80 ± 0.02	10.00 ± 0.02

**Fig. 16:**
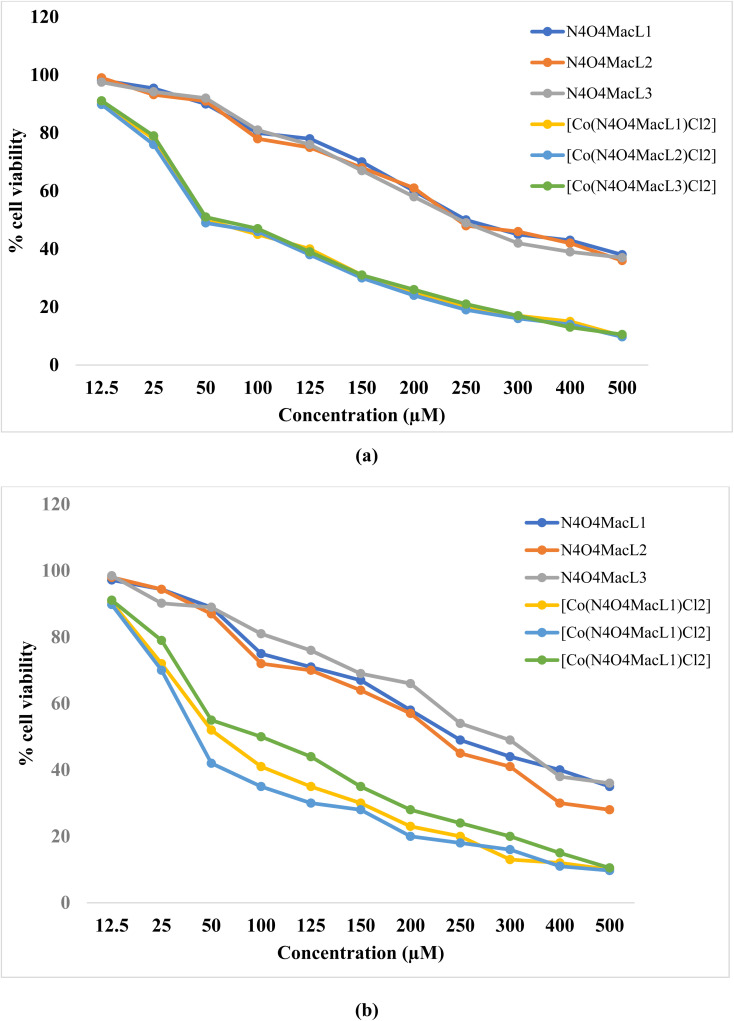
Dose-dependent survival profiles of (a) MCF-7 and (b) HepG-2 cancer cell lines.

Importantly, the observed cytotoxic activity correlates well with predictions from molecular docking studies.^69,70^ The colorimetric viability assays further indicate that both the ligands and their Co(ii) complexes exhibit cytotoxic effects against MCF-7 cells comparable to colchicine (Fig. 15), with Co(ii) complexes showing higher average levels of relative cell inhibition. Fig. 16 depicts the survival curves of the cancer cell lines treated with the synthesized compounds.

### 3.11 Computational molecular docking analysis

**Fig. 17:**
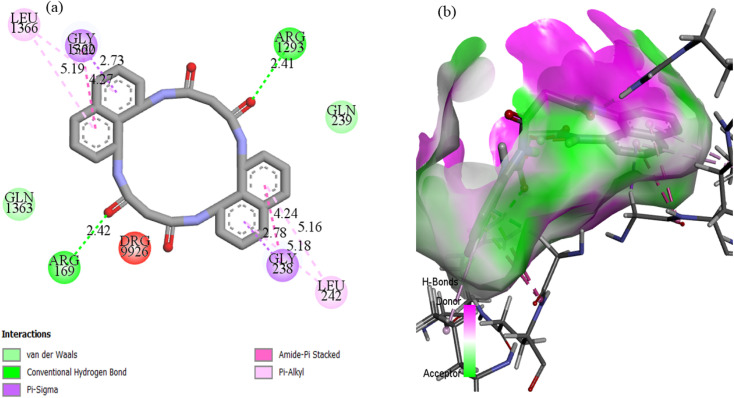
(a) Two-dimensional and three-dimensional interaction profiles of N_4_O_4_MacL_1_ with the *E. coli* protein receptor.

To investigate the protein-binding potential of the synthesized ligands computationally, molecular docking studies were performed to evaluate their interaction with various receptor proteins. These studies provided binding affinities and inhibitory constants, indicating that most ligands exhibited significant binding potential. Docking simulations were carried out using the AutoDock Vina module within PyRx software.^71^ Protein structures in PDB format (3T88, 3TY7, 3DRA, 5H67) were loaded as N_4_O_4_Macromolecules, while ligand preparation involved generating different tautomer's, assigning bond orders, defining ring conformations, and specifying stereochemistry. These targets were chosen based on their relevance to the pathogenic organisms tested in our antimicrobial studies and their biological role in essential cellular pathways, such as cell wall synthesis, enzyme regulation, nucleic acid processing, and oxidative stress response. Each protein represents a clinically significant therapeutic target associated with resistance development or virulence. Furthermore, previously reported studies have demonstrated the involvement of these proteins in pathogen survival, making them suitable receptors for molecular docking to evaluate potential inhibitory interactions of the synthesized macrocycles and metal complexes (Fig. 17).^72^

**Fig. 18:**
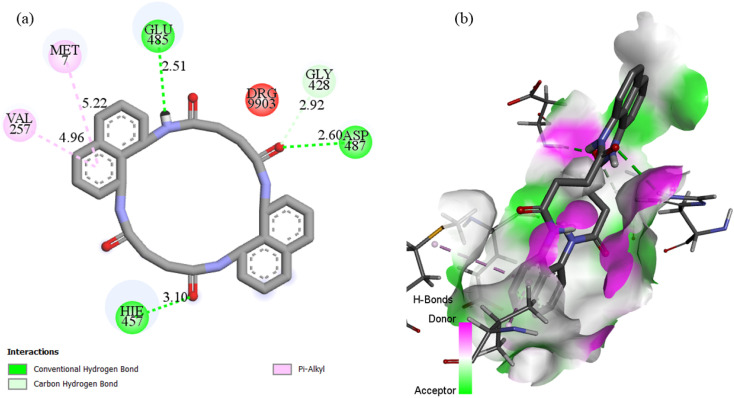
(a) Two-dimensional and three-dimensional interaction profiles of N_4_O_4_MacL_1_ with the 3row protein receptor.

For *Escherichia coli*, the crystal structure 3T88 (ref. 73) was employed for docking, which is critical for assessing protein function related to cell growth and viability. The best-docked conformation of ligand N_4_O_4_MacL_1_ exhibited a binding score of −12.8 kcal mol^−1^ (Table 14), indicating strong inhibition. The ligand formed two conventional hydrogen bonds with ARG169 and ARG1293, with bond lengths of 2.41 Å and 2.42 Å, respectively (Fig. 18). Docking of N_4_O_4_MacL_1_–N_4_O_4_MacL_3_ ligands yielded minimal binding energies of −12.8, −10.3, and −11.5 kcal mol^−1^, respectively, highlighting N_4_O_4_MacL_1_ as the ligand with the strongest predicted interaction.

**Table 14:** Computational molecular docking results of the synthesized macrocyclic compounds

Compounds	Active protein (PDB ID)	Binding affinity (kcal mol^−1^)	Number of H-bond	Binding mode
[N_4_O_4_MacL_1_]	3T88	−12.8	2	ARG′169, AGG′1293
	3ROW	−9.9	1	ALA′497
	3DRA	−9.8	1	TYR′550
	5H67	−9.6	3	ARG′54, ARG′45, SER′142
[N_4_O_4_MacL_2_]	3T88	−10.3	1	ARG′60
	3ROW	−9.7	4	ASP′487, GLU′485,GLY428, HIE′457
	3DRA	−11.3	2	ARG′615, HIE′425
	5H67	−10.8	3	GLU′50, LYS′135, SER′52
[N_4_O_4_MacL_3_]	3T88	−11.5	1	ARG′341
	3ROW	−10.3	3	GLU′103, ASN′627, THR′859
	3DRA	−11.2	2	MET′624, PRO′623
	5H67	−9.7	1	GLU′50

**Fig. 19:**
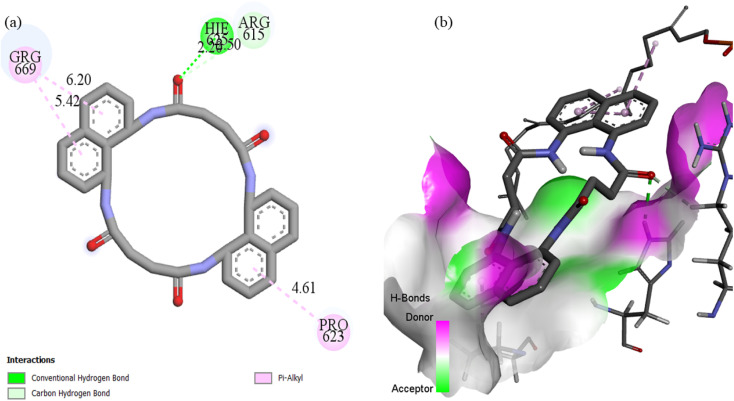
(a) Two-dimensional and three-dimensional interaction profiles of N_4_O_4_MacL_2_ with the 3dra protein receptor.

**Fig. 20:**
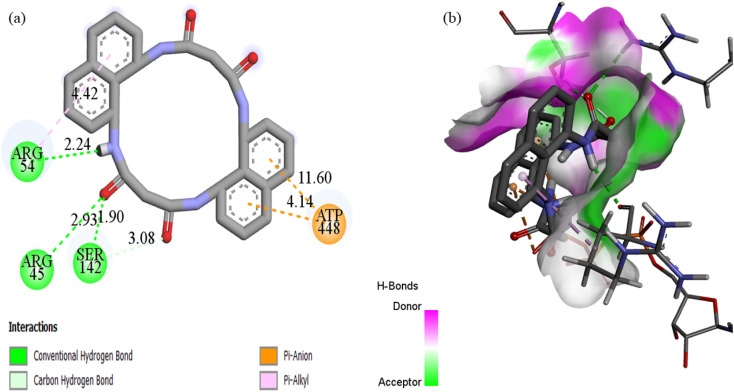
(a) Two-dimensional and three-dimensional interaction profiles of N_4_O_4_MacL_1_ with the 5h67 protein receptor.

Docking with the N4O4Macromolecule 3ROW revealed hydrogen bonding interactions of the ligands with GLU485, ASP487, HIE457, and GLY428,^74^ at bond distances of 2.50, 2.59, 3.01, and 2.89 Å, respectively (Fig. 18). The corresponding binding energies for N_4_O_4_MacL_1_–N_4_O_4_MacL_3_ were −9.8, −9.8, and −10.4 kcal mol^−1^, with N_4_O_4_MacL_3_ showing the strongest negative binding energy, indicating a more favorable interaction with the protein. Similar docking analyses were performed for PDB structures 3DRA and 5H67, as depicted in Fig. 19 and 20. Overall, these docking results correlate well with experimental observations from spectroscopic studies, supporting the proposed groove-binding mode of the ligands to the target biomolecules.

## 4 Conclusion

The Co(ii) complex derived from 1,8-diaminonaphthalene was successfully synthesized and characterized using a combination of analytical and multi-spectroscopic techniques, confirming its biochemical and physiological activity. FT-IR analysis indicated coordination through the amide nitrogen atoms, validating ligand donation to the central metal. The proposed distorted octahedral geometry of the Co(ii) complex was aadditionally validated through complementary UV-visible, ESI-MS, and EPR spectroscopic evidence. DFT calculations corroborated the predicted structures of the synthesized compounds. *In vitro* cytotoxicity studies demonstrated that [Co(N_4_O_4_MacL_2_)Cl_2_] and [Co(N_4_O_4_MacL_1_)Cl_2_] showed pronounced anticancer efficacy against MCF-7 cells, yielding IC_50_ values of 19.35 ± 0.23 µM and 21.54 ± 0.30 µM, respectively. In addition, the synthesized compounds demonstrated significant antimicrobial potential, showing effective inhibition against both bacterial and fungal pathogens. Molecular docking studies revealed favorable binding energies, suggesting potential biological targets for the complexes. Specifically, [Co(N_4_O_4_MacL_3_)Cl_2_] exhibited strong antimicrobial activity, showing a minimum inhibitory concentration of 67 µg mL^−1^ toward *Candida albicans*. In addition, antioxidant studies demonstrated excellent radical-quenching efficiency for [Co(N_4_O_4_MacL_2_)Cl_2_], reflected by an IC_50_ value of 35.08 ± 1.22 µg mL^−1^. Docking simulations further indicated that the Macrocyclic ligand N_4_O_4_MacL_3_ displayed higher negative binding affinities toward receptors 3T88, 3DRA, 3ROW, and 5H67, supporting the observed biological activities. These results collectively suggest that the amide-based ligands and their Co(ii) complexes are promising candidates for antimicrobial, anticancer, and antioxidant applications.

## Author contributions

The research concept was proposed by Subhash and Ashu Chaudhary. Experimental work was carried out by Manish Kumar and Vandana. Subhash performed data analysis, manuscript preparation, and editing. All authors critically reviewed and approved the final manuscript.

## Conflicts of interest

The authors declare no competing interests.

## Supplementary Material

RA-016-D5RA08873A-s001

## Data Availability

All data are available in the article and its supplementary information (SI). Supplementary information is available. See DOI: https://doi.org/10.1039/d5ra08873a.

## References

[cit1] Ferlay J., Shin H. R., Bray F., Forman D., Mathers C., Parkin D. M. (2010). Int. J. Cancer.

[cit2] Kumar S., Devi J., Dubey A., Kumar D., Jindal D. K., Asija S., Sharma A. (2023). Res. Chem. Intermed..

[cit3] Erdmann A., Menon Y., Gros C., Masson V., Aussagues Y., Ausseil F., Novosad N., Schambel P., Baltas M., Arimondo P. B. (2016). Future Med. Chem..

[cit4] Khalid W., Badshah A., UllahKhan A., Nadeem H., Ahmed S. (2018). Chem. Cent. J..

[cit5] Gaber M., Khedr A. M., Elsharkawy M. (2017). Appl. Organomet. Chem..

[cit6] Alka A., Gautam S., Kumar R., Singh P., Gandhi N., Jain P. (2023). Results Chem..

[cit7] Chandrasekhar V. R., Palsamy K. M., Lokesh R., Thangadurai D., Gandhi I. N., Jegathalaprathaban R., Gurusamy R. (2019). Appl Organometal Chem..

[cit8] Kargara H., Behjatmanesh-Ardakanib R., Torabib V., Sarvianb A., Kazemic Z., Chavoshpour-Natanzic Z., Mirkhanic V., Sahraeid A., Tahire M. N., Ashfaq M. (2021). Inorg. Chim. Acta.

[cit9] Liu X., Hamon J. R. (2019). Coord. Chem. Rev..

[cit10] Sharma N., Dhingra N., Choudhary G., Chauhan S., Singh H. L. (2025). Integrated Spectroscopic, Computational, and Antimicrobial Profiling of Phenylsilicon (IV) Schiff Base Complexes. J. Indian Chem. Soc..

[cit11] Ghosh D., Choudhury S. T., Ghosh S., Mandal A. K., Sarkar S., Ghosh A., Saha K. D., Das N. (2012). Chem Biol Interact.

[cit12] Jafari M., Salehi M., Kubicki M., Arab A., Khaleghian A. (2017). Inorg. Chim. Acta.

[cit13] Mohamed A. A., Ahmed F. M., Zordok W. A., El-Shwiniy W. H., Sadeek S. A., Elshafe H. S. (2022). Inorganics.

[cit14] El-Attar M. S., Ahmed F. M., Sadeek S. A., Mohamed S. F., Zordok W. A., El-Shwiniy W. H. (2022). Appl. Organomet. Chem..

[cit15] Sharma N., Dhingra N., Singh H. L. (2024). Design, spectral, antibacterial and in-silico studies of new thiosemicarbazones and semicarbazones derived from symmetrical chalcones. J. Mol. Struct..

[cit16] Wani W. A., Malik A. H., Hussain A., Alajmi M. F., Shreaz S., Kostova I. (2025). Recent advances in metal complexes for the photodynamic therapy of cancer. New J. Chem..

[cit17] El-Boraey H. A., Aly S. A. (2013). Syn. React. Inorg. Met..

[cit18] Cocu M., Bulhac I., Coropceanu E., Melnic E., Shova S., Ciobanica O., Gutium V., Bourosh P. (2014). J. Mol. Struct..

[cit19] Rawat P., Singh R. N., Ranjan A., Ahmad S., Saxena R. (2017). Spectrochim. Acta.

[cit20] Karadeniz S., Yuksektepe Ataol C., Ozen T., Demir R., Ogütçü H., Bati H. (2019). J. Mol. Struct..

[cit21] Wani R. J., Hussain A., Sheikh M. U. D., Bukhari M. N., Fatima M., Ali G. (2025). G. Synthesis of mono and hetero-bi nuclear lanthanum (III) and europium (III) complexes of 2-hydroxy-5-sulfobenzoic acid and 1, 10-phenanthroline: luminescence and magnetic studies. J. Mater. Sci. Mater. Eng..

[cit22] Jeragh B., Ali M. S., El-Asmy A. A. (2015). Spectrochim. Acta.

[cit23] Burgos-Lopez Y., Del Pla J., Balsaa L. M., Leon I. E., Echeverri G. A., Piro O. E., Garcia-Tojal J., Pis-Diez R., Gonzalez-Baro A. C., Parajon-Costa B. S. (2019). Inorg. Chim. Acta.

[cit24] Bhaskar R., Salunkhe N., Yaul A., Aswar A. (2015). Spectrochim, Acta.

[cit25] Subhash J., Chaudhary A. (2023). Res. Chem. Intermed..

[cit26] Subhash A., Chaudhary J., Kumar M., Solanki R. (2023). J. Iran. Chem. Soc..

[cit27] Khodiev M., Holikulov U., Jumabaev A., Issaoui N., Lvovich L. N., Al-Dossary O. M., Bousiakoug L. G. (2023). J. Mol. Liq..

[cit28] Mahmoud W. H., Deghadi R. G., Mohamed G. G. (2016). J. Therm. Anal. Calorim..

[cit29] Rudbari H. A., Iravani M. R., Moazam V., Askari B., Khorshidifard M., Habibi N., Bruno G. (2016). J. Mol. Struct..

[cit30] Singh H. L., Dhingra N., Bhanuka S. (2023). Synthesis, spectral, antibacterial and QSAR studies of tin and silicon complexes with Schiff base of amino acids. J. Mol. Struct..

[cit31] Wani W. A. , Alajmi M. F., Hussain A., and Wani I. A., Therapeutic Potential of Gold Complexes, Royal Society of Chemistry. 2025

[cit32] Bose D. S., Idrees M., Jakka N. M., Rao J. V. (2010). J. Comb. Chem..

[cit33] Abu-Dief A. M., Abdel-Rahman L. H., Abdel-Mawgoud A. A. H. (2020). Appl. Organomet. Chem..

[cit34] El-Metwaly N., Katouah H., Aljuhani E., Alharbi A., Alkhatib F., Aljohani M., Alzahrani S., Alfaifi M. Y., Khedr A. M. (2020). J. Inorg. Organomet. Polym. Mater..

[cit35] Morris G. M., Huey R., Lindstrom W., Sanner M. F., Belew R. K., Goodsell D. S., Olson A. J. (2009). J. Comput. Chem..

[cit36] Subhash S., Chaudhary A., Mamta M., Jyoti M. (2023). Chem. Pap..

[cit37] Giffin G. A., Moretti A., Jeong S., Passerini S. (2014). J. Phys. Chem. C.

[cit38] Al-Gaber M. A. I., Abd El-Lateef H. M., Khalaf M. M., Shaaban S., Shawky M., Mohamed G., Abdou A., Gouda M., Abu-Dief A. M. (2023). Materials.

[cit39] Abdou A., Mostafa H. M., Abdel-Mawgoud A. M. M. (2022). Inorg. Chim. Acta.

[cit40] Malhotra M., Rawal R. K., Malhotra D., Dhingra R., Deep A., Sharma P. C. (2017). Arab. J. Chem..

[cit41] Mishra M., Tiwari K., Shukla S., Mishra R., Singh V. P. (2014). Spectrochim. Acta A Mol. Biomol. Spectrosc..

[cit42] Purandara H., Forob S., Gowdaa B. T. (2015). Acta. Crystallogr. E..

[cit43] Mahmoud W. H., Mohamed G. G., El-Dessouky M. M. I. (2014). Int. J. Electrochem. Sci..

[cit44] Manikshete A. H., Awatade M. M., Sarsamkar S. K., Asabe M. R. (2015). Int. J. Eng. Sci. Inv..

[cit45] West D. X., Yang Y., Klein T. L., Goldberg K. I., Liberta A. E., Valdes Martinez J., Hernandez-Ortega S. (1995). Polyhedron.

[cit46] Fataftah M. S., Krzyaniak M. D., Vlaisavljevich B., Wasielewski M. R., Zadrozny J. M., Freedman D. E. (2019). Chem. Sci..

[cit47] Chandra S., Gautam A., Tyagi M. (2007). J. Trans Met chem..

[cit48] Kopel P., Travnicek Z., Marek J., Korabik M., Mrozinski J. (2003). Polyhedron.

[cit49] Kavitha N., Anantha Lakshmi P. V. J. (2017). Saudi Chem. Soc..

[cit50] Coats A. W., Redfern J. P. (1964). Nature.

[cit51] Horowitz H. W., Metzger G. (1963). Anal. Chem..

[cit52] Jyothi S., Sreedhar K., Nagavaju D., Swamy S. J. (2015). Can Chem Trans..

[cit53] Tyagi M., Chandra S. (2012). Open J. Inorg. Chem..

[cit54] Koley M. K., Chouhan O. P., Biswas S., Fernandes J., Banerjee A., Chattopadhyay A., Varghese B., Manoharan P. T., Koley A. P. (2017). Inorg. Chim. Acta.

[cit55] Alghuwainem Y. A., Abd El-Lateef H. M., Khalaf M. M., Abdelhamid A. A., Alfarsi A., Gouda M., Abdou A. (2023). J. Mol. Liq..

[cit56] Kumar M., Darolia P. J., Chauhan S., Sindhu M., Verma K. K., Garg S. (2021). ChemistrySelect.

[cit57] Subhash S., Phor A., Chaudhary A., Jyoti M. (2024). J. Inorg. Organomet. Polym..

[cit58] Kumar M., Darolia P. J., Chauhan S., Sindhu M., Verma K. K., Garg S. (2021). ChemistrySelect.

[cit59] Mahmoud W. H., Mahmoud N. F., Mohamed G. G. (2017). Applied Organomet. Chem.

[cit60] Mahmoud W. H., Mohamed G. G., Refat A. M. (2017). Applied Organomet..

[cit61] Raman N., Raja J. D., Sakthivel A. (2007). J. Chem. Sci..

[cit62] Latif M. A., Ahmed T., Hossain M. S., Chaki B. M., Abdou A., Kudrat-E-Zahan M. (2023). Russ. J. Gen. Chem..

[cit63] Subhash A., Chaudhary J., Kumar M., Kumar N., Agarwal N. K. (2022). J. Chem. Sci..

[cit64] Dharmaraj N., Viswanathamurthi P., Natarajan K. (2001). Transition Met. Chem..

[cit65] Akki M., Reddy D. S., Katagi K. S., Kumar A., Devarajegowda H. C., Babagond V., Mane S., Joshi S. D. (2022). J. Mol. Struct..

[cit66] Gull P., Malik M. A., Dar O. A., Hashmi A. A. (2017). Microb. Pathog..

[cit67] Rathi P., Singh D. P. (2015). J. Mol. Struct..

[cit68] Haribabu J., Subhashree G. R., Saranya S., Gomathi K., Karvembu R., Gayathri D. (2015). J. Mol. Struct..

[cit69] Ebrahimipour S. Y., Sheikhshoaie I., Castro J., Haase W., Mohamadi M., Foro S., Sheikhshoaie M., Mahani S. E. A. (2015). Inorg. Chim. Acta.

[cit70] Sharma A., Dhingra N., Singh H. L., Khaturia S., Bhardawaj U. (2022). New Complexes of organotin (IV) and organosilicon (IV) with 2-{(3, 4-dimethoxybenzylidene) amino}-benzenethiol: Synthesis, spectral, theoretical, antibacterial, docking studies. J. Mol. Struct..

[cit71] Subhash A., Chaudhary J., Kumar M., Solanki R. (2023). J. Iran. Chem. Soc..

[cit72] Arafath M. A., Adam F., Ahamed M. B. K., Karim M. R., Uddin M. N., Yamin B. M., Abdou A. (2023). J. Mol. Struct..

[cit73] Elshakre M. E., Mahmoud A., Noamaan H., Butt Moustafa H. (2020). Int. J. Mol. Sci..

[cit74] Kumar A., Bora U. (2014). Interdiscip. Sci. Comput. Life Sci..

