# Supplementary material for: Novel amide-based macrocyclic Co(ii) complexes: correlating structural, computational, and biological properties through DFT and docking
Source: RSC Adv. 2026 Jan 14;16(4):3169–91. doi: 10.1039/d5ra08873a (PMC12803015; doi:10.1039/d5ra08873a)
Supplement: RA-016-D5RA08873A-s001 [file RA-016-D5RA08873A-s001.pdf]

Supplementary Information (SI) for RSC Advances.
This journal is © The Royal Society of Chemistry 2025

SUPPLEMENTRY INFORMATION

Novel Amide-Based Macrocyclic Co(II) Complexes: Correlating Structural, Computational, and Biological Properties Through DFT and Docking

Subhash[a], [c], Manish Kumar[b], Vandana[c], Annu Devi[c], and Ashu Chaudhary*[c]

[a] Department of Chemistry, Smt. Devkiba Mohansinhji Chauhan College of Commerce and Science, Silvassa-396230, DNH, India
[b] Department of Chemistry, Maharshi Dayanand University-124001, Haryana, India
[c] Department of Chemistry, Kurukshetra University, Kurukshetra-136119, Haryana, India
E-mail: ashuchaudhary@kuk.ac.in

Table of contents

Sr. No.	Content	Page No.
1	FTIR of Macrocyclic ligand $N_4O_4MacL_2$, $N_4O_4MacL_3$ and complex (9b) and (9c)	2-3
2	Mass Spectrum of Macrocyclic ligand $N_4O_4MacL_1$- $N_4O_4MacL_3$	3-4
3	Mass Spectrum of Co(II) complex $[Co(N_4O_4MacL_1)Cl_2]$ (9a)	4
4	Mass spectrum of Co(II) complex $[Co(N_4O_4MacL_3)Cl_2]$ (9c).	5
5	The linear fitted curve obtained through the Coats-Redfern method FWO method at 10°/min heating rate of the ligands and their Co(II) macrocyclic complexes.	5-7
7	Physical properties and analytical data of Macrocyclic ligands and Co(II) complexes	8
8	EPR parameters of Co(II) complexes	9

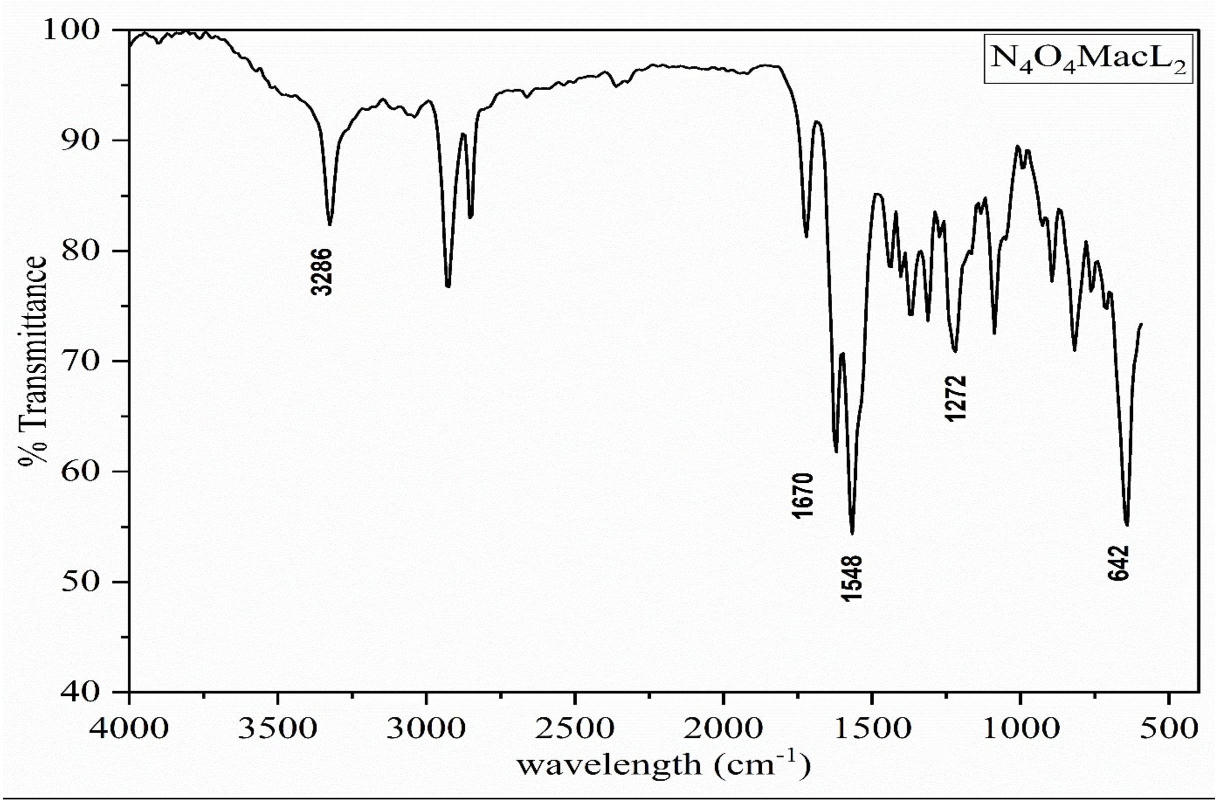

FIGURE S1 FTIR spectra of Macrocyclic ligand $N_4O_4ML_2$

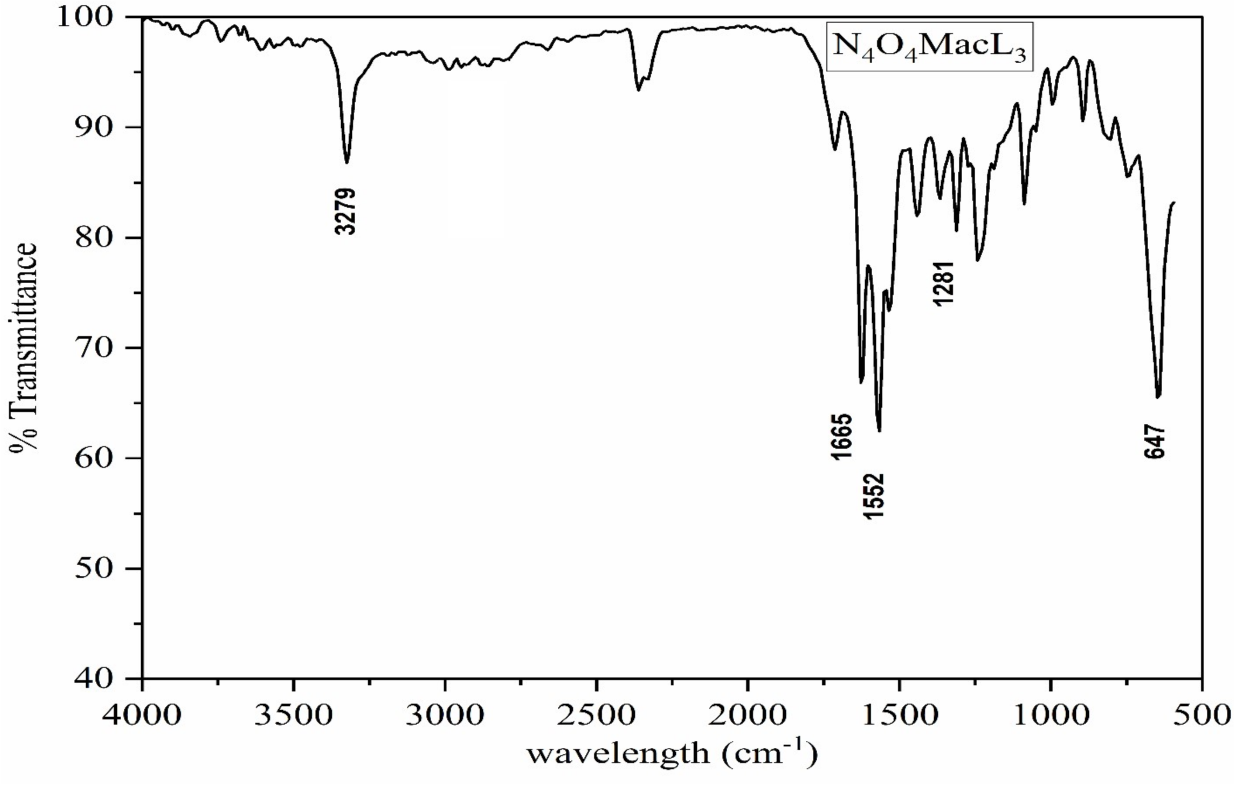

FIGURE S2 FTIR spectra of tetraamide macrocyclic ligand $N_4O_4ML_3$

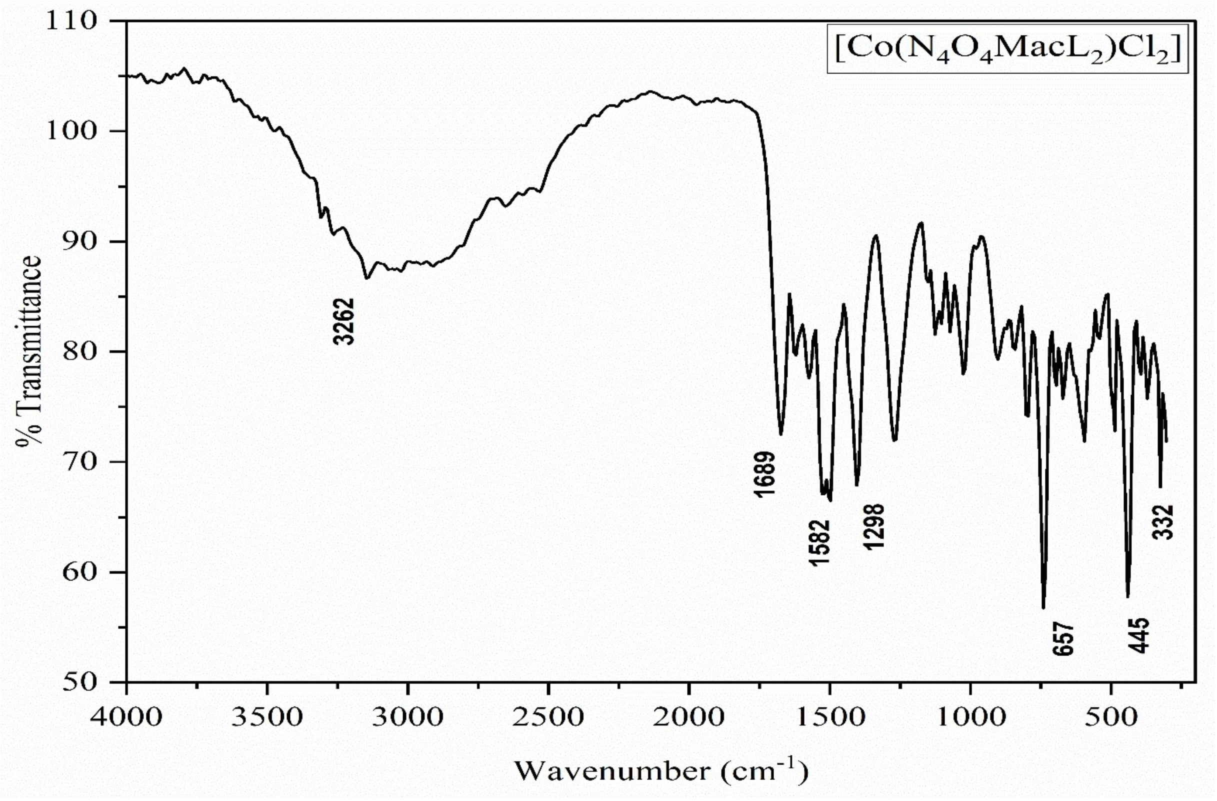

FIGURE S3 FTIR spectra of cobalt(II) Macrocyclic Complex $[Co(N_4O_4ML_2)Cl_2]$ (8b).

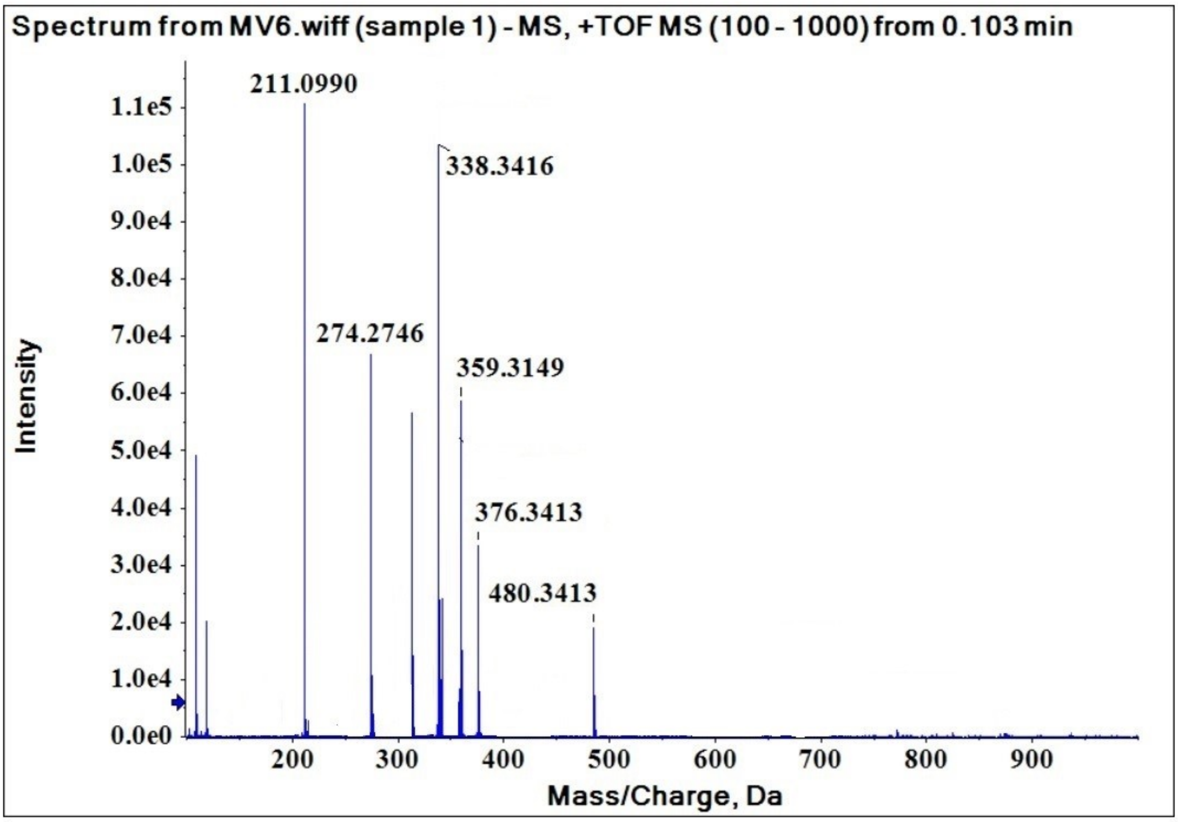

FIGURE S4 Mass spectrum of tetraamide Macrocyclic ligand $N_4O_4MacL_1$

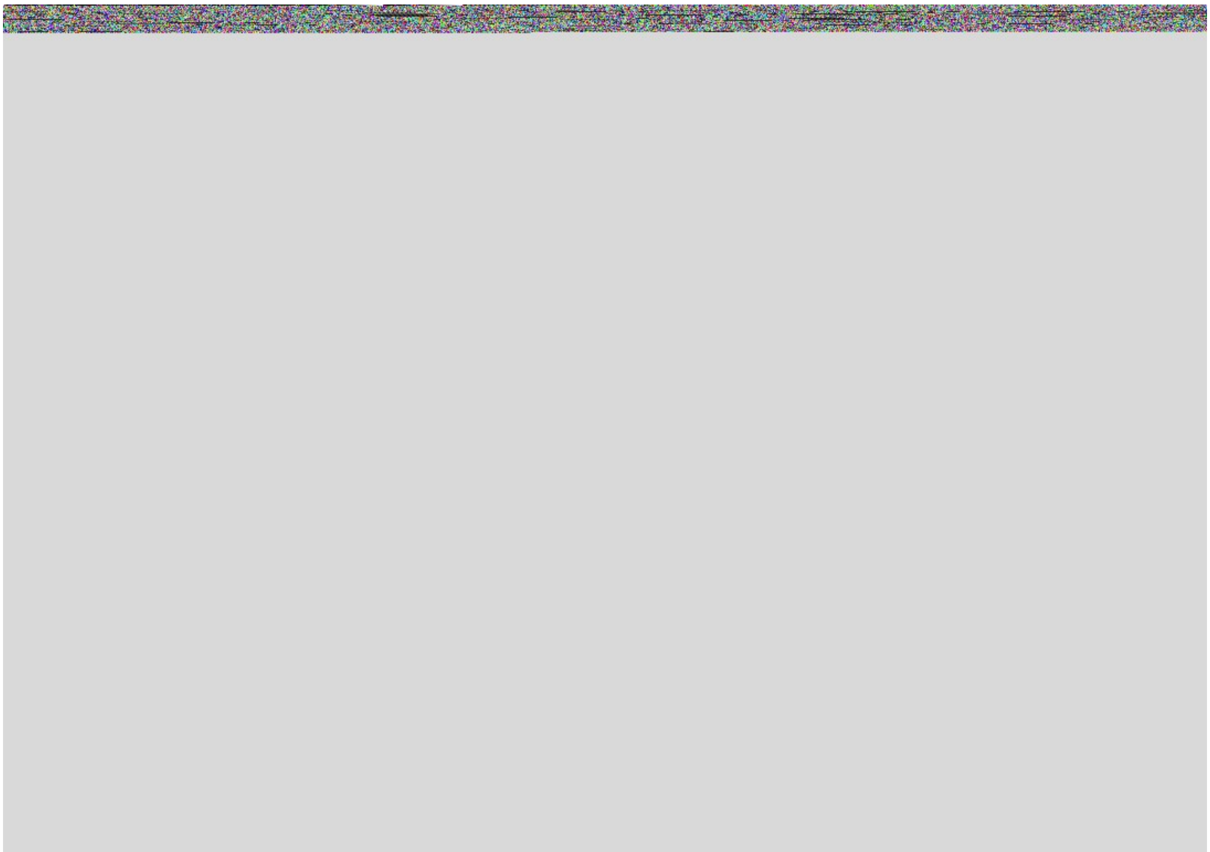

FIGURE S5 Mass spectrum of Co (II) Macrocyclic Complex $[Co(N_4O_4ML_2)Cl_2]$

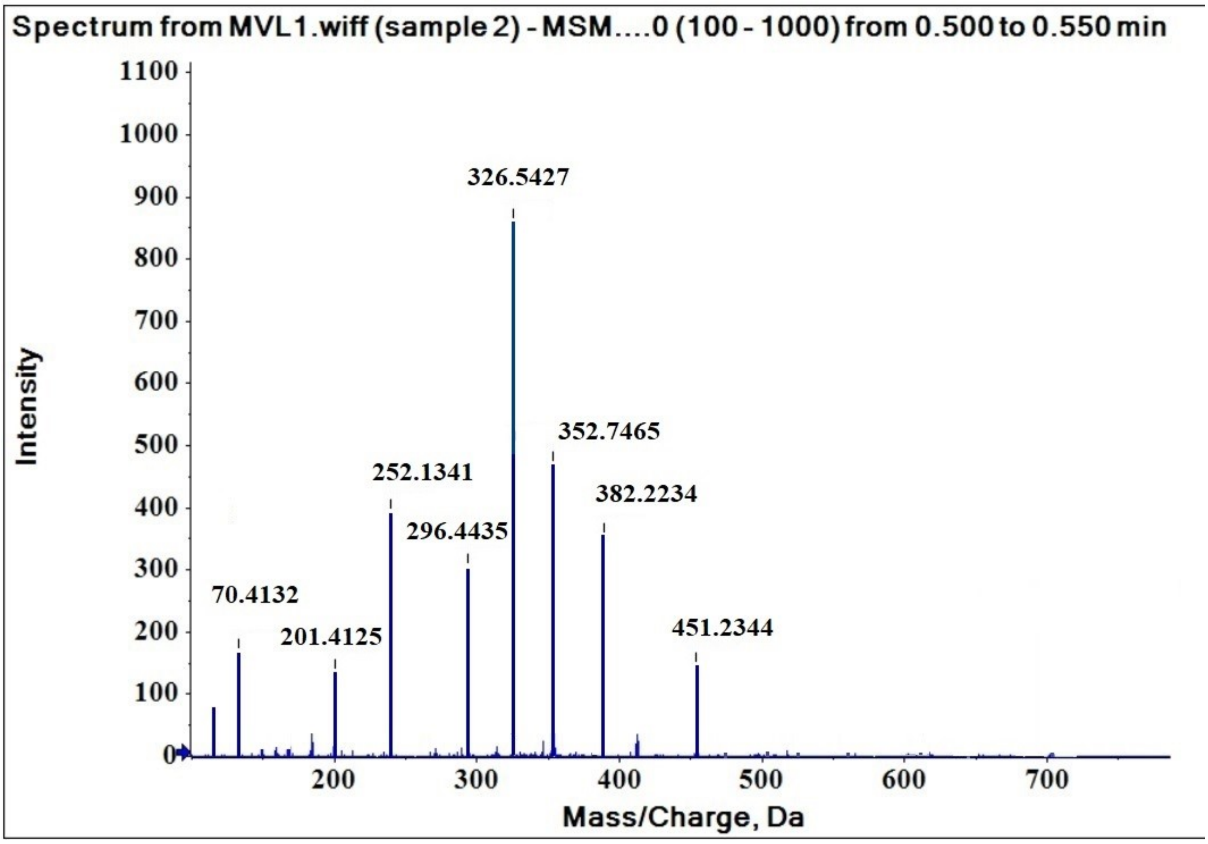

FIGURE S6 Mass spectrum of tetraamide Macrocyclic ligand $N_4O_4MacL_1$

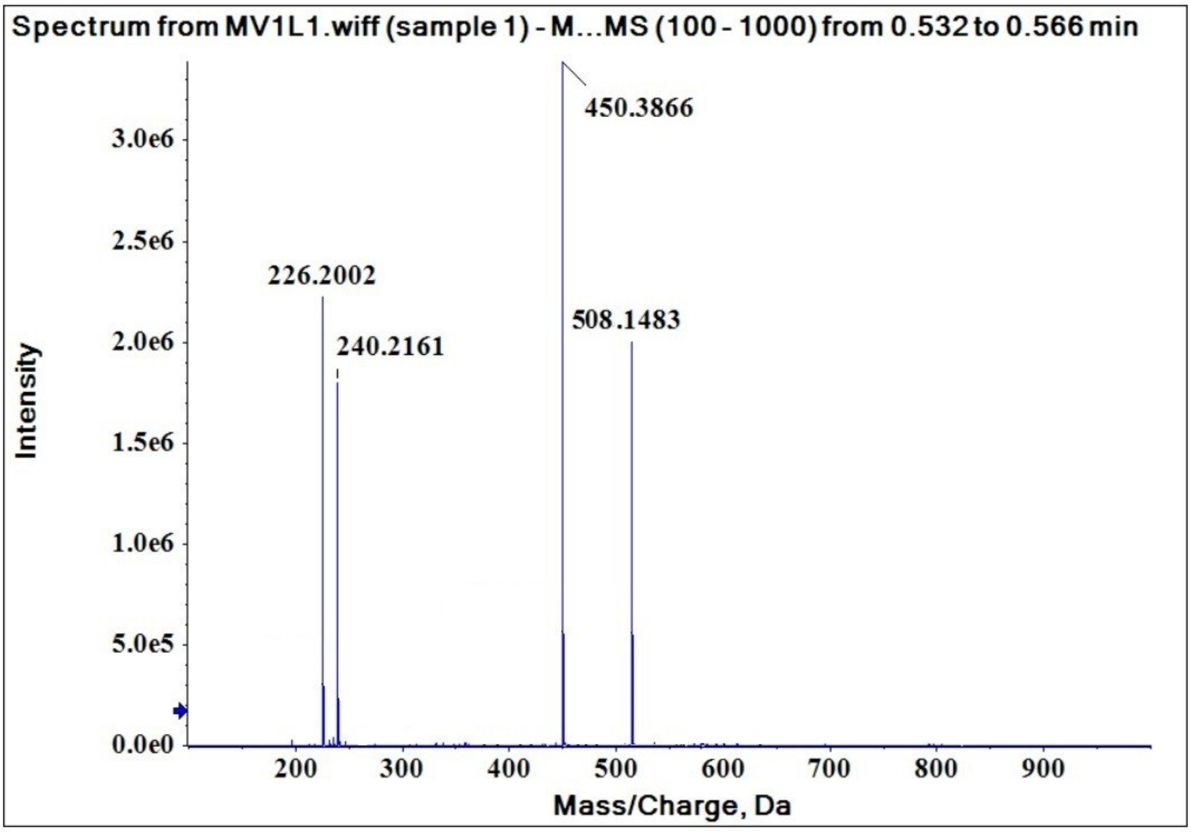

FIGURE S7 Mass spectrum of tetraamide Macrocyclic ligand $N_4O_4MacL_3$

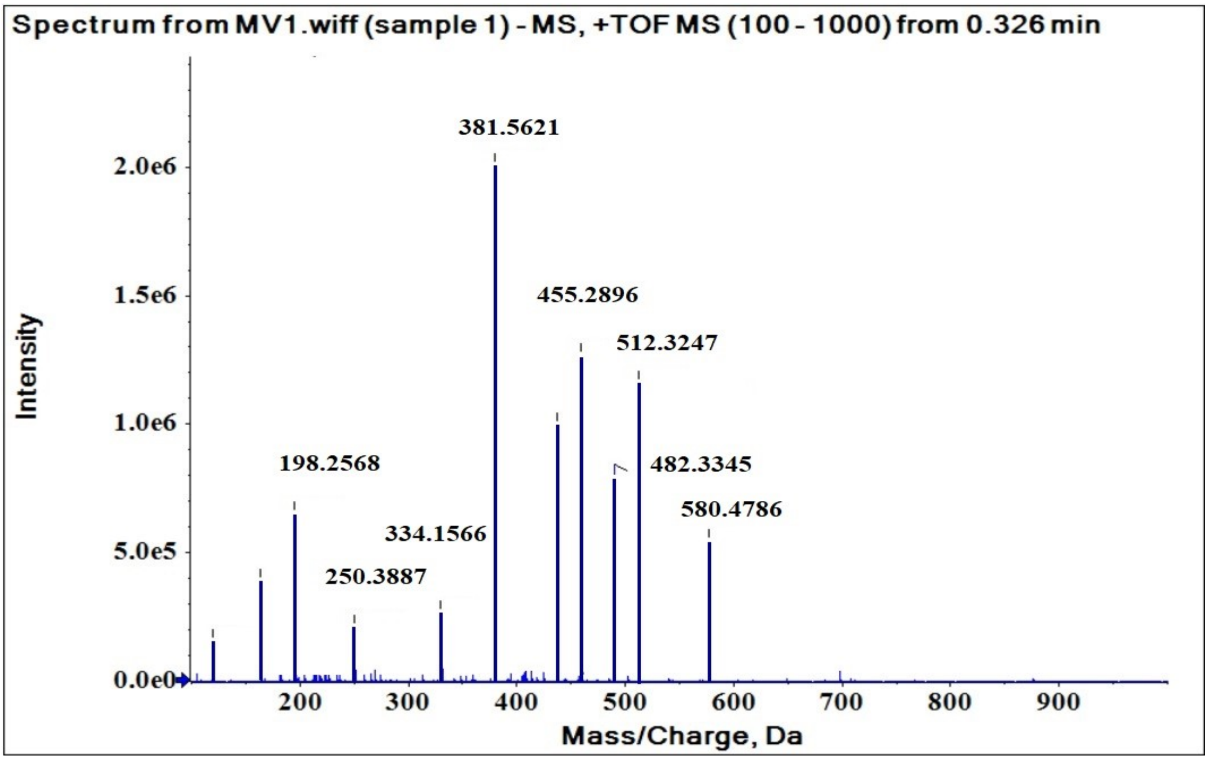

FIGURE S8 Mass spectrum of Co (II) Macrocyclic Complex [Co($N_4O_4ML_2$)Cl_2]

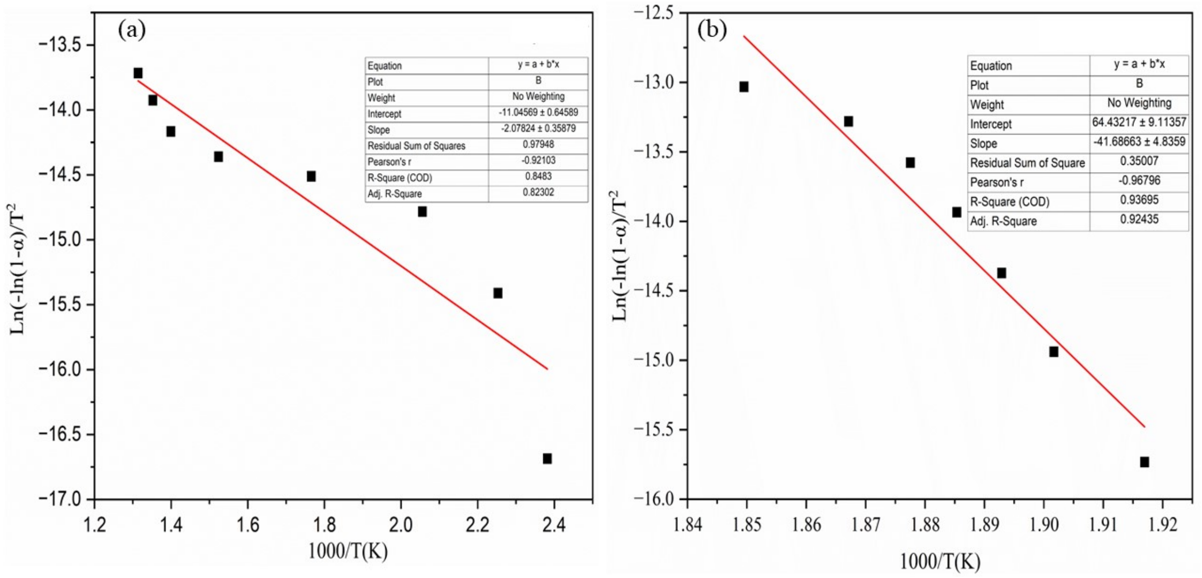

FIGURE S9 Coats-Redfern plots at 10°/min heating rate for macrocyclic ligand $N_4O_4MacL_2$ and complex [Co($N_4O_4MacL_2$)Cl_2]

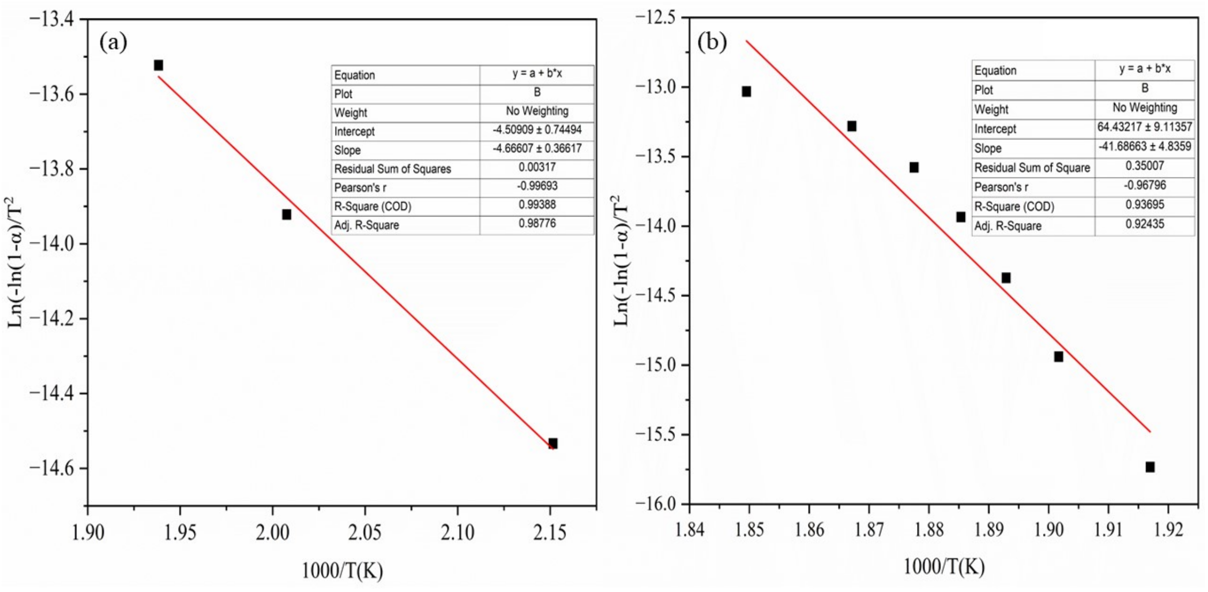

FIGURE S10 Coats-Redfern plots at 10°/min heating rate for macrocyclic ligand $N_4O_4MacL_3$ and complex $[Co(N_4O_4MacL_3)Cl_2]$.

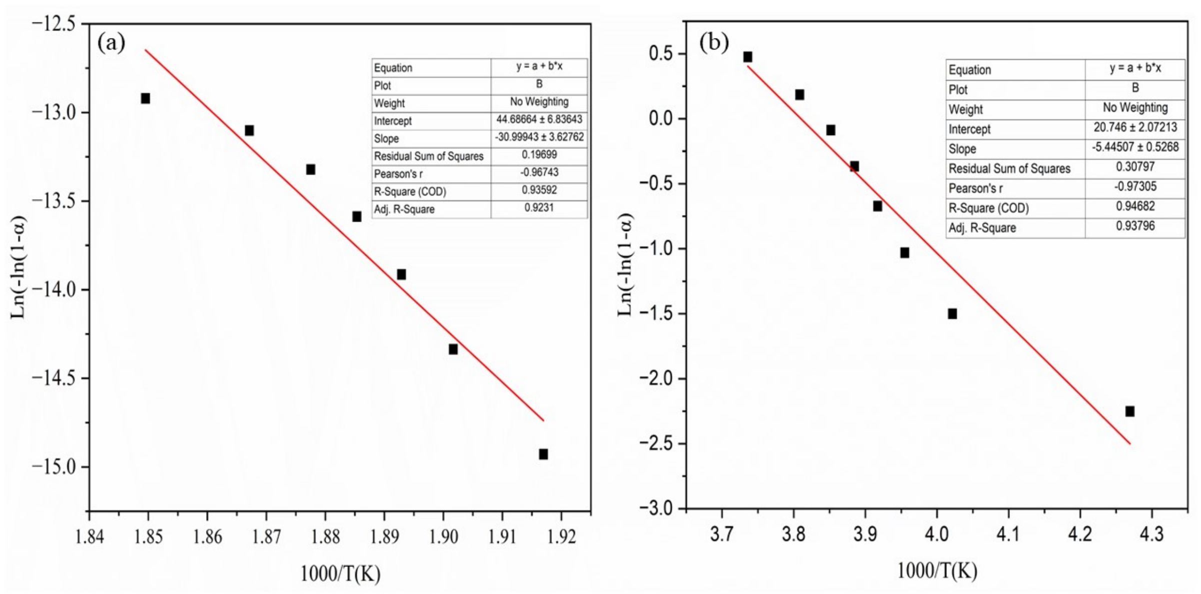

FIGURE S11 The linear fitted curve obtained through FWO method at 10°/min heating rate of macrocyclic ligand $N_4O_4MacL_2$ and Complex $[Co(N_4O_4MacL_2)Cl_2]$

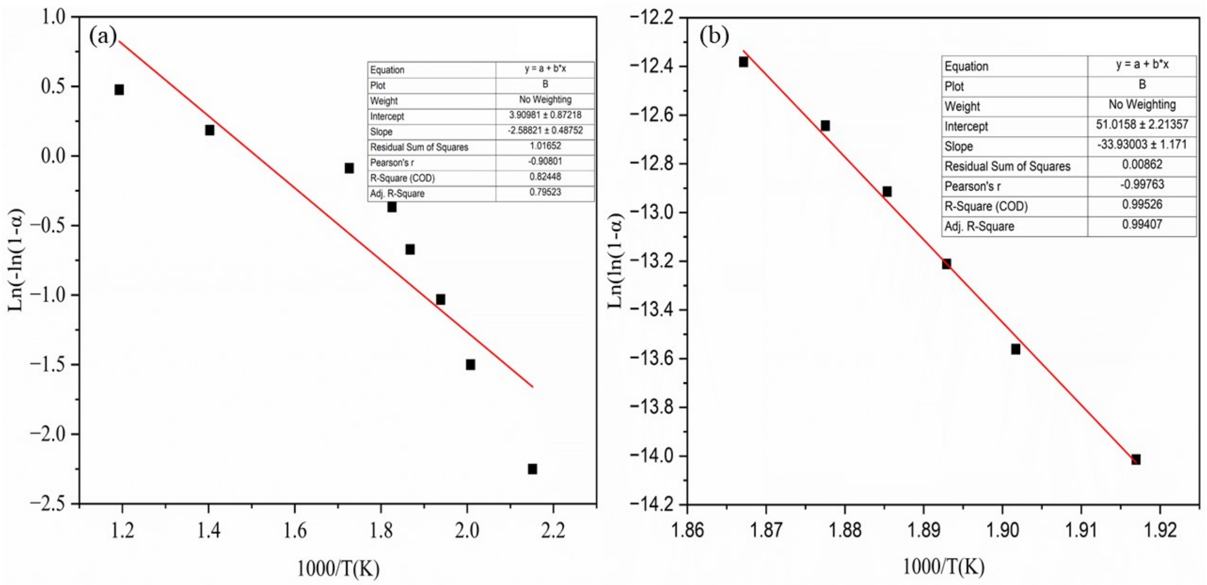

FIGURE S12 The linear fitted curve obtained through FWO method at 10°/min heating rate of macrocyclic ligand $N_4O_4MacL_3$ and Complex $[Co(N_4O_4MacL_3)Cl_2]$

TABLE S1 Physical properties and analytical data of macrocyclic ligands and their Co(II) complexes

Compound	Empirical Formula	% Yield	M.P.^{0}C & Colour	Analysis, Found (Calcd.) %					Mol. Wt. Found (Calcd.)
				C	H	N	Cl	Co	
$N_4O_4MacL_1$	$\mathbf{C_{26}H_{20}N_4O_4}$	75	220-225 Dark Brown	68.45 (69.02)	4.43 (4.46)	12.12 (12.38)	-	-	451.44 (452.47)
$N_4O_4MacL_2$	$\mathbf{C_{28}H_{24}N_4O_4}$	79	215-221 Brown	68.94 (69.99)	5.08 (5.03)	11.28 (11.66)	-	-	480.3413 (480.52)
$N_4O_4MacL_3$	$\mathbf{C_{30}H_{28}N_4O_4}$	78	225-232 Brown	70.65 (70.85)	5.37 (5.55)	11.10 (11.02)	-	-	508.14 (508.21)
$[Co(N_4O_4MacL_1)Cl_2]$	$\mathbf{C_{26}H_{20}Cl_2CoN_4O_4}$	77	245-250 Reddish Pink	53.54 (53.63)	3.38 (3.46)	9.46 (9.62)	12.11 (12.18)	10.02 (10.12)	580.47 (582.32)
$[Co(N_4O_4MacL_2)Cl_2]$	$\mathbf{C_{28}H_{24}Cl_2CoN_4O_4}$	71	252-257 Brown	55.05 (55.10)	3.79 (3.96)	9.10 (9.18)	11.45 (11.62)	9.59 (9.66)	608.61 (610.36)
$[Co(N_4O_4MacL_3)Cl_2]$	$\mathbf{C_{30}H_{28}Cl_2CoN_4O_4}$	80	255-259 Brown	56.35 (56.44	4.32 (4.42)	08.70 (08.78)	11.05 (11.11)	9.18 (9.23)	638.61 (638.41)

Table S2. EPR parameters of Co(II) complexes

Complexes	$g_{\parallel}$	$g_{\perp}$	|g|	G	$A_{\parallel} \times 10^5 (cm^{-1})$	$K^2_{\parallel}$	$K^2_{\perp}$	-λ
$[Co(N_4O_4MacL_1)Cl_2]$	2.24	2.07	2.12	4.31	605	0.593	0.556	496
$[Co(N_4O_4MacL_2)Cl_2]$	2.25	2.05	2.12	4.29	605	0.594	0.554	499
$[Co(N_4O_4MacL_3)Cl_2]$	2.29	2.04	2.12	4.24	604	0.596	0.556	497
